# Improving genome-scale metabolic models of incomplete genomes with deep learning

**DOI:** 10.1016/j.isci.2024.111349

**Published:** 2024-11-07

**Authors:** Meine D. Boer, Chrats Melkonian, Haris Zafeiropoulos, Andreas F. Haas, Daniel R. Garza, Bas E. Dutilh

**Affiliations:** 1 Theoretical Biology and Bioinformatics, Utrecht University, 3584 CH Utrecht, the Netherlands; 2 Department Marine Microbiology and Biogeochemistry, NIOZ Royal Netherlands Institute for Sea Research, PO Box 59, Den Burg 1790 AB, Texel, The Netherlands; 3 Bioinformatics Group, Wageningen University and Research, Wageningen, the Netherlands; 4 Laboratory of Molecular Bacteriology, Rega Institute for Medical Research, Department of Microbiology, Immunology and Transplantation, KU Leuven, 3000 Leuven, Belgium; 5 Université Paris-Saclay, INRAE, PROSE, 92761 Antony, France; 6 Institute of Biodiversity, Faculty of Biological Sciences, Cluster of Excellence Balance of the Microverse, Friedrich Schiller University Jena, 07743 Jena, Germany

**Keywords:** Microbial genomics, Biocomputational method, Computational Bioinformatics, Genomic analysis

## Abstract

Deciphering microbial metabolism is essential for understanding ecosystem functions. Genome-scale metabolic models (GSMMs) predict metabolic traits from genomic data, but constructing GSMMs for uncultured bacteria is challenging due to incomplete metagenome-assembled genomes, resulting in many gaps. We introduce the deep neural network guided imputation of reactomes (DNNGIOR), which uses AI to improve gap-filling by learning from the presence and absence of metabolic reactions across diverse bacterial genomes. Key factors for prediction accuracy are: (1) reaction frequency across all bacteria and (2) phylogenetic distance of the query to the training genomes. DNNGIOR predictions achieve an average F1 score of 0.85 for reactions present in over 30% of training genomes. DNNGIOR guided gap-filling was 14 times more accurate for draft reconstructions and 2–9 times for curated models than unweighted gap-filling.

## Highlights

• We trained a deep neural network on >11k bacterial species to recover missing reactions
• Reaction frequency and query similarity to the training data impacted performance
• DNNGIOR models can simulate real data similar to CarveMe with fewer false positives

## Graphical abstract

**Figure:**
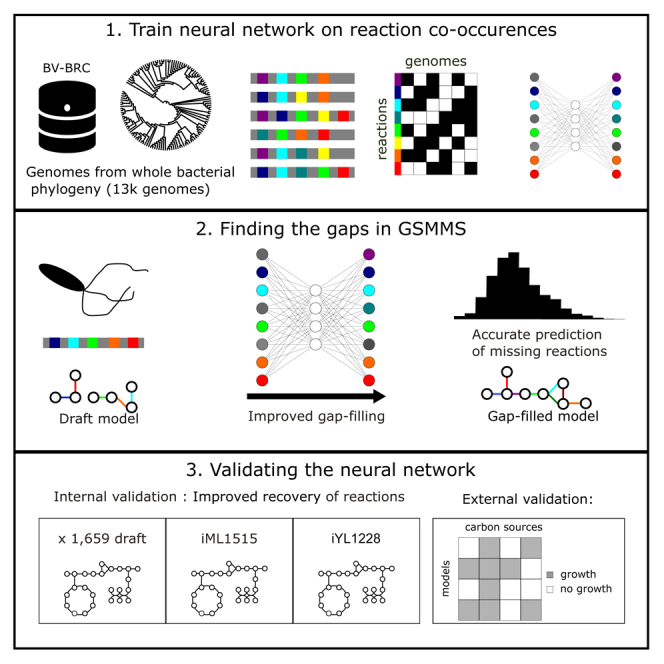

## Introduction

Simulating microbial metabolism is an effective method to understand bacterial physiology and interactions within their communities.^1^^,^^2^^,^^3^ The functions and interactions of bacteria can be inferred from their genome sequences using genome-scale metabolic models (GSMMs).^3^^,^^4^^,^^5^^,^^6^ GSMMs can be constructed either manually or automatically with tools such as Cobrapy,^7^ RAVEN,^8^ ModelSEED,^9^ KBase,^10^ and CarveMe,^11^ which identify metabolic reactions encoded on the genome and build a metabolic network. However, if the original genome sequence is incomplete, a common occurrence with metagenome-assembled genomes (MAGs), the inferred GSMM will also be incomplete.^12^ Consequently, gaps in GSMMs emerge due to missing knowledge and errors introduced during sequencing,^13^ binning,^14^^,^^15^ and annotation.^16^ In the past, gap-filling was primarily executed through manual curation,^17^^,^^18^^,^^19^^,^^20^ but this method is time-consuming and does not scale well for studies that include a large number of GSMMs.^21^^,^^22^^,^^23^^,^^24^

Several algorithms have been developed to automate gap-filling, such as FastGapfilling,^25^ GlobalFit,^26^ CHESHIRE,^27^ OptFill,^28^ and DEMETER^29^ that add reactions that allow a GSMM to simulate growth or match phenotypic profiles. The reaction sets that can gap-fill a model are not unique^30^ and the organism’s actual metabolism may not always align with the minimal set of reactions satisfying a user-defined objective.^31^ This indicates room for refining gap-filling algorithms to yield more realistic solutions. As most gap-filling algorithms allow us to weight reactions individually according to their likelihood of being in the model,^11^^,^^25^^,^^30^ several attempts have been made to find weights based on genomics,^32^ proteomics,^8^ topology,^27^^,^^30^ or reaction type.^9^^,^^33^ Nevertheless, despite these advances, determining the optimal weights for any reaction and any model still remains challenging.^4^ The limitations of the currently employed methods and their limited usage of phylogenetic information as a signal for gap-filling, opens up an opportunity for a machine-learning based approach to better optimize these weights.

In this article, we introduce DNNGIOR: a Python package that uses a neural network to assign weights to metabolic reactions to complete GSMMs that are built from incomplete genomes. This neural network is trained to discern patterns in the co-occurrences of reactions across the bacterial domain and to predict reactions based on incomplete reaction sets, with the goal of assessing which reactions may be missing from an incomplete network. This information will be useful for automated and manual GSMM reconstruction. When we used the predictions of the neural network to weight reactions in incomplete GSMMs, we found that the accuracy of the neural network depends on the frequency of reactions in the training data and the phylogenetic relatedness of genomes used to generate these data. We benchmarked the predictions using both automated and manually curated models, including data from a recent study on carbon usage profiles by plant-associated bacteria.^34^

## Results

**Figure 1:**
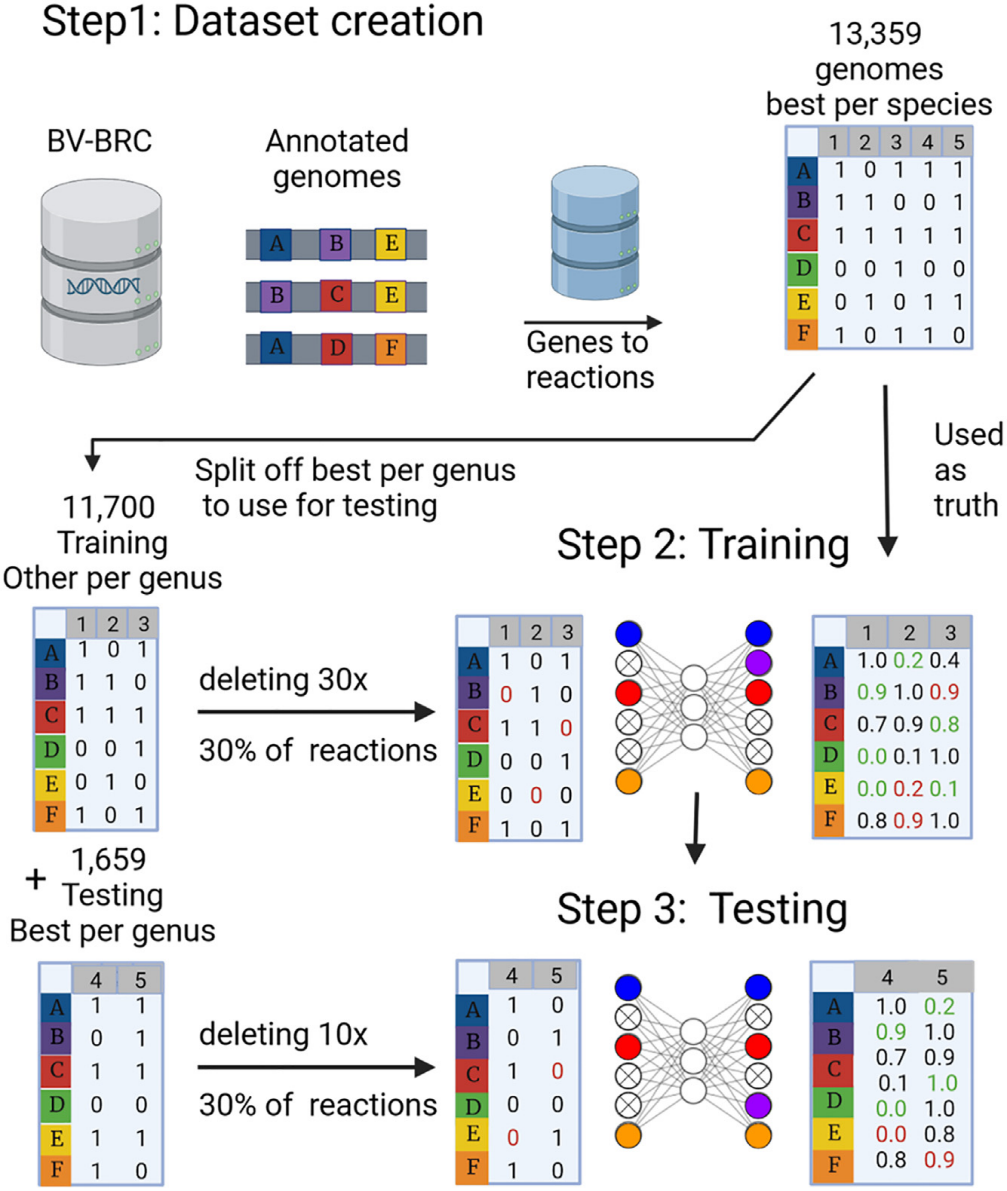
Schematic overview of training and testing the DNNGIOR neural network Step 1: constructing the dataset. Genomes were collected from the BV-BRC database^35^ selecting one genome per species (13,359 genomes), genomes were annotated and metabolic networks constructed as outlined in STAR Methods section “Collection and processing of training and testing datasets”. The resulting incidence matrix of reactions in different genomes was split into subsets for testing (best per genus, 1,659 genomes) and training (remaining 11,700 genomes). Step 2: training the neural network. We randomly deleted 30% of reactions 30x to simulate incomplete genomes. The network was trained to predict which reactions were removed, while not predicting the reactions that were not part of the original draft model. All reactions that were given as input to the network are ignored when calculating the loss from the predictions. Step 3: testing the neural network. To estimate the prediction accuracy for different reactions in a diverse set of organisms, the network was tested on the 1,659 genomes in the testing dataset. Incomplete genomes were simulated as above, 10x per genome. This figure was created using BioRender.^36^

We set out to build a tool that would make use of the co-occurrence patterns of reactions found in a broad range of bacterial genomes to improve the reconstruction of metabolic models from incomplete genomes and MAGs. Therefore, we trained a neural network on the occurrence of 2,457 or 4,240 metabolic reactions for ModelSEED and CarveMe, respectively, in over 13 thousand species (training and testing datasets). This network predicts missing reactions within incomplete reaction sets. A schematic overview of our approach is depicted in Figure 1.

### Reaction frequency is an important factor for prediction accuracy

Before testing the predictions to guide gap-filling we first wanted to understand the factors underlying accurate predictions by the DNNGIOR neural network. Understanding these factors can show the strengths of the network, show possible areas of improvement, and provide new insights into the gap-filling problem. We identified two important factors that affect the accuracy of predictions by the neural network: (1) the frequency of reaction across all bacteria and (2) the phylogenetic distance between the organisms in the testing and training dataset.

**Figure 2:**
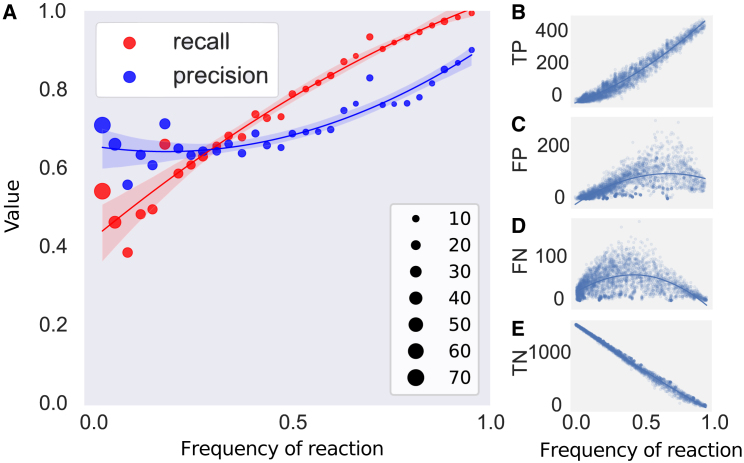
Prediction accuracy increases with reaction frequency (A) Relationship between the recall (red) and precision (blue) of predictions as a function of the reaction frequency (the fraction of models in the testing dataset where a reaction occurs). Reactions were binned in ranges of 50 models, dot size corresponds to number of reactions in the bin, the y-axis shows precision and recall. The shaded region shows the 95% confidence interval of the regression. Recall = TP/(TP + FN), Precision = TP/(TP + FP). (B–E) Regression plots of True Positives (TP, B), False Positives (FP, C), False Negatives (FN, D) and True Negatives (TN, E) as a function of reaction frequency (the fraction of models in the testing dataset where a reaction occurs). Trend lines were estimated using a polynomial regression model. A ROC curve of neural network predictions can be found in Figure S2. For more metrics and standard deviations see Table S1.

Frequent reactions have higher recall and precision than rare reactions, with core reactions that are present in >90% of all bacteria having a recall of 0.96 (sd = 0.007) and precision of 0.86 (sd = 0.039) (Figure 2A). The neural network has more opportunities to learn reactions and their associated patterns when they are common than when a reaction is only present in a few genomes.

Specifically, true positives (TPs) increase linearly with frequency (Figure 2B) while true negatives (TN) decrease linearly (Figure 2E). Since the total number of times a reaction is deleted also increases with frequency, there are more opportunities to correctly predict that a reaction is present or absent. Moreover, the false positives (FPs) and false negatives (FNs) decrease for more frequent reactions (Figures 2C and 2D). This indicates that the neural network can accurately predict whether a reaction should be present or absent from the model if they are sufficiently represented in the training dataset. This makes it important to approach predictions for rare reactions cautiously, as they are more likely to be incorrect. However, as these reactions make an organism and their metabolic role in communities unique, we still feel that they should be considered.

### Training data can be adapted to reflect the frequency of missing reactions in real data

As we have seen previously, the frequently occurring reactions can be more accurately predicted. However, the most frequent reactions might not be the ones that are most likely to be missing from incomplete models in practice. Several biases in binning and annotation affect the distribution of missing reactions in real data. For instance, accessory genes are more likely to be missing from MAGs because the binning of mobile genetic elements is less effective than the rest of the genome.^15^ Additionally, large genomes with many accessory genes are more difficult to annotate than smaller genomes with mostly core genes.^16^ As accessory genes are by definition rarer than core genes,^37^ this leads to a bias toward rare genes being missing from MAGs. Another important bias is that taxa with less-researched members are more difficult to annotate accurately using homology-based tools.^16^^,^^38^ Finally, reactions can be perceived to be rarer because they are more often missing from the data.

These biases could be a concern if we want to use DNNGIOR to gap-fill models based on larger genomes, MAGs, or from the less researched parts of the bacterial domain as reactions missing from those models are more likely to be rare. Therefore, we tested the DNNGIOR neural network on data that contained a deliberate deletion bias, where rarer reactions were deleted more often (see Equations 1a and 1b in the STAR Methods). In this case, the F1-score decreased 36% as the network overestimated common reactions and underestimated rare reactions (Figure S1). In contrast, if the network was trained on data with the same bias toward deleting rarer reactions, this effect was reduced (from 36 to 18%), and the network became better at predicting rare reactions (Figure S1). This means that it is possible to train the network to account for biases that may be present in the data. However, given that the bias in the data are variable and difficult to quantify, DNNGIOR uses a neural network trained on uniform deletion by default, as will all further analysis unless specifically mentioned otherwise.

### Short phylogenetic distances and complete representation improve prediction

**Figure 3:**
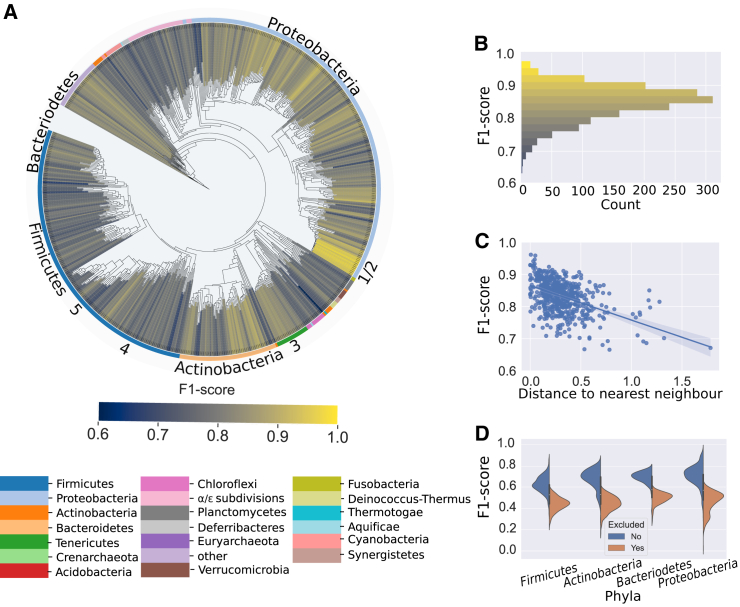
Phylogenetic distance influences the accuracy of DNNGIOR neural network predictions (A) Phylogenetic tree based on a concatenated multiple sequence alignment of 71 single-copy marker genes of all the 1,659 genomes in the testing dataset. Branch color represents the F1-score, the color of the outer ring corresponds to the phyla ordered by size, all phyla with less than five species are combined in other, the four largest phyla are annotated. The five species with high-quality curated metabolic models used during the gap-fill analysis are also marked: 1. *Escherichia coli,* 2. *Klebsilla pneumoniae*, 3. *Synechococcus elongatus*, 4. *Bacillus subtilis*, and 5. *Streptococcus aureus*. The tree is based on a concatenated alignment of hits to HMM profile of 71 single-copy marker genes. Phylogenetic distances in the tree represent the number of amino acid substitutions per site. (B) Histogram of the F1-scores colored with the same color map as the Tree. For more metrics and standard deviations see Table S1. (C) Scatterplot of the F1-score versus the distance to the nearest neighbor expressed in the Jaccard distance, trendline (Pearson r^2^ = −0.51, *p* = 1.53∗10^−40^). (D) Split violin plots of F1-scores of the predictions for neural networks when different phyla are included (left) or excluded (right) from the training dataset. Interior of the violin shows the median and the interquartile range. All differences between splits were significant (Table S2).

We wanted to determine next which genomes are predicted better. We expected that predictions would be better for genomes from well-sampled taxa than from taxa with only few sequenced relatives. To test this, we plotted the F1-score for all genomes in the testing dataset on a phylogenetic tree (Figure 3A). While the DNNGIOR neural network scored well on most models (mean F1-score = 0.84, sd = 0.054, Figure 3B), predictions were more accurate for species that had close relatives in the training dataset than for species that were more distantly related. When we made predictions for every species in the testing dataset, we found that the F1-score of the predictions correlated with the distance to the closest neighbor (Pearson r^2^ = 0.261, *p* = 1.53∗10^−40^). Indeed, species that have close relatives in the training dataset are easier to predict than species that are phylogenetically unique (Figure 3C).

The correlation between F1-score and interspecies distance illustrates the importance of a good and complete representation of the bacterial domain as this would reduce the average distance between query species and those in the training dataset. We confirmed this hypothesis by excluding some phyla from the training dataset, which resulted in lower F1-scores compared to when all phyla were included (Figure 3D). We also confirmed that the reverse is true, i.e., training on only reaction sets from one phylum increases the performance of that phylum (Figure S3). Using the training module, DNNGIOR neural networks can be trained on any collection of genomes, e.g., from a certain taxonomic group or environmental biome. This sacrifices general applicability, but can improve the performance (Figure S4). Several specialized networks are available on GitHub for this purpose.

### Neural network weights improve gap-filling of draft models

**Figure 4:**
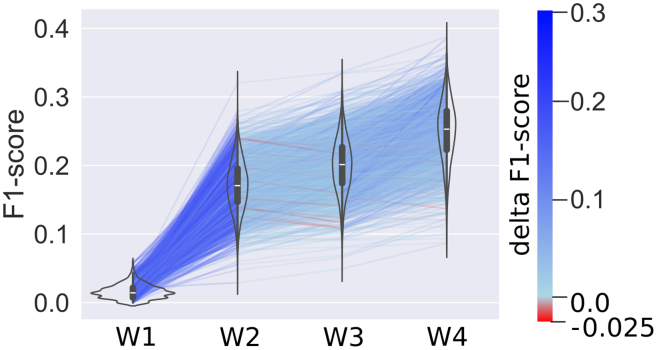
Weighted gap-filling of draft models Violin plots of F1-scores of the gap-filling of 1,659 models in the testing dataset, from which we randomly deleted 30% of reactions in triplicate. These reduced models were gap-filled using four different weighting schemes (Equation 4). For W1 (“No weights”) all reactions in the database are weighted equally. For W2 (“Naive binary weights”) all reactions that are present in the training dataset were given the same weights. For W3 (“Frequency-based weights”) the frequency of the reaction was used to weight reactions. For W4 (“NN-weights”) the prediction scores generated by the DNNGIOR neural network were used. Lines connect the same models to show trends and are colored based on the difference in F1-score for the model between weighting schemes. Interior of the violin plots shows the median and the interquartile range. All groups were significantly different (Table S3). Violin Plots of recall, precision, TP, FP, FN, and TNs can be found in Figure S5. For mean F1-scores and standard deviations see Table S4.

Next, we determined the effectiveness of using NN-predicted weights to guide gap-filling, compared to other alternatives. For this, we gap-filled the models from the testing dataset using the half-interval gap-filling algorithm (see STAR Methods), where weights may be assigned to individual reactions and the algorithm finds a metabolic network that is capable of generating a biomass flux, while minimizing the weights that are added overall. We assessed whether the artificially removed reactions could be recovered when reactions were weighted based on four different weighting schemes: W1. No weights, W2. Naive binary weights, W3. Frequency-based weights, and W4. NN-weights (see STAR Methods). To be clear, the process of gap-filling results in a functional model that can produce biomass, while the training dataset consists of draft models that generally cannot produce biomass and contain exclusively reactions derived from genome annotations, from which a fraction was deleted (see Figure 1). We found that NN-weights (W4) significantly improved the accuracy of automated gap-filling compared to other weighting schemes (Table S3), i.e., they allowed a larger fraction of the deleted reactions to be recovered in the model. By using scheme W4, the F1-score increased by a factor of 13.98 times compared to W1 (*p* = 4∗10^−18^), 1.92 times compared to W2 (*p* = 4∗10^−18^), and 1.09 times compared to W3 (*p* = 3∗10^−18^, Figure 4).

When we try to explain the improvement observed, a major part is already visible with W2 which shows that the reactions from the pan-reactome are indeed the most important ones. The improvement with W3 is in line with the observations from assessing the accuracy of the predictions of the DNNGIOR neural network directly, namely that frequency of a reaction is also important for gap-filling. Although this may be expected, the reaction frequency has often been neglected when gap-filling strategies are developed. These strategies often focus on flux or network topology, giving the same cost to all reactions. This leads to addition of a minimum number of reactions that are necessary for growth, agnostic to all external information. Here, we found that simply weighing reactions by their frequencies in the bacterial domain already significantly improves the accuracy of gap-filling (Figure 4). The DNNGIOR neural network scores (W4) further improve gap-filling, suggesting that additional information has been learned from the co-occurrence patterns.

When observing these results, we note that the F1-scores were lower than might be expected based on the prediction accuracy (Figure 2B). This consistent trend can be attributed to the fact that the draft models were already incomplete before the additional reactions were removed, because they were based on genome annotations alone. Thus, some reactions were necessarily added by the half-interval gap-filling algorithm to enable biomass production that was counted as FPs here since they were not present in the genome annotation. Furthermore, the objective of the half-interval gap-filling algorithm was not to find back reactions but rather to find a set of reactions that allows biomass production, while minimizing their overall weights. As many of the annotated reactions that were removed were not strictly required for biomass production they were not added back, leading to FNs. Although the F1-scores were thus systematically reduced, we can still interpret the trends in performance of the four different weighting schemes in Figure 4.

Overall, reactions that were assigned a high probability by the DNNGIOR neural network are likely to be present in the metabolic network, whereas low probability reactions are likely to be absent. To further incentivize the inclusion of high probability reactions while still satisfying the biomass production objective, we provided these reactions with negative, rather than zero costs (see Equation 2). These reactions were thus even more stimulated to be included, and indeed this nearly doubled (x1.95) the F1-score compared to using only positive weights (Figure S6A). However, this approach also led to an increase in FPs for reactions that were absent but were still assigned high probabilities by DNNGIOR (Figure S6B).

The testing of gap-filling against the AGORA2 model collection^22^ showed the same pattern but overall lower scores (Figure S7), presumably because the gap-filling algorithm does not generally find reactions that are non-essential for growth, while those reactions may have been added during curation by DEMETER^29^ as part of the AGORA2 pipeline.

### Neural network-based weights improve gap-filling of curated models

**Figure 5:**
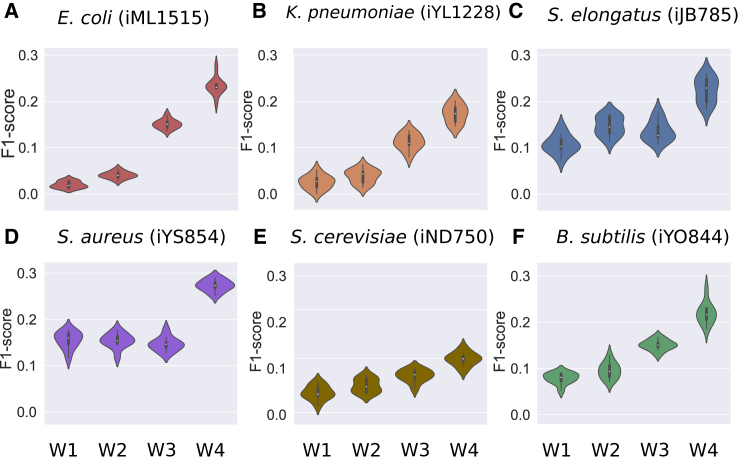
Weighted gap-filling of six curated metabolic models Violin plots of F1-scores of the gap-filling of curated models, from which we randomly deleted 30% of reactions 10 times. These reduced models were gap-filled using four different weighting schemes (Equation 4). For W1 (“No weights”) all reactions in the database are weighted equally. For W2 (“Naive binary weights”) all reactions that are present in the training data were given the same weights. For W3 (“Frequency-based weights”) the frequency of the reaction was used to weight reactions. For W4 (“NN-weights”) the prediction scores generated by the DNNGIOR neural network were used. (A–E) (A) *E. coli* iML1515, (B) *K. pneumoniae* iYL1228, (C) *S. elongatus* iJB785, (D) *S. aureus* iYS854, (E) *S. cerevisiae* iND750, and (F) *B. subtilis* iYO844. Interior of the violin shows the median and the interquartile range.

Next, we assessed the performance of the four weighting schemes using six high-quality manually curated models. As previously, we artificially removed reactions from these models and tested how well these reactions were reintroduced when weighted by the DNNGIOR neural network (see STAR Methods). As shown in Figure 5, the NN-weights (W4) outperformed the other weights for all six models. Notably, *Saccharomyces cerevisiae* (iND750) performed the worst of the six tested models (mean = 0.11, sd = 0.05). This may be expected since *S. cerevisiae* is a eukaryote and only distantly related to the prokaryotic reference genomes that comprised the DNNGIOR training dataset. The best performance (mean = 0.22, sd = 0.08) was found for *Escherichia coli* (iML1515), a species from a well-studied family with many reference genomes. The good performance of the *E. coli* model, but also of the other models might in part be explained by the fact that they are derived from relatively well-studied taxa with extensive annotation. Thus, these models perform better than most draft models in our testing dataset previous. We also performed a test where only reactions that were deemed essential were removed, showing that scores improved (Figures S8A–S8F). Notably, this also revealed a bias where more frequent reactions were more often essential (Figures S8G and S8H), especially in *K. pneumoniae* (iYL1228) and *S. elongatus* (iJB785). This led to reduced performance of NN-weights (W4) compared to frequency-based weights (W3).

**Figure 6:**
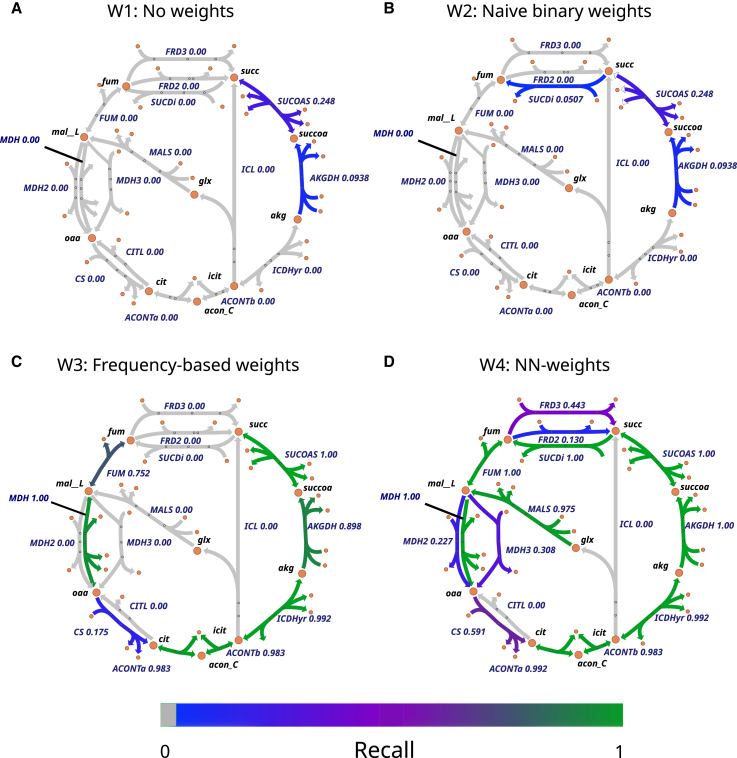
Recall of the reactions in the citric acid cycle of *E. coli* Escher maps of the citric acid cycle colored by the mean recall after gap-filling models, from which we randomly deleted 30% of the reactions 500 times. (A–D) Reduced models were gap-filled using four different weighting schemes: (A) W1: No weights (B) W2: Naive binary weights (C) W3: Frequency-based weights and (D) W4: NN-weights (see Figure 5 caption and STAR Methods for details). IDs for secondary metabolites were omitted, the full central metabolism can be found in (Figures S10 and S11). For the gap-filling scores of the 500 duplicates of the *E. coli* model using different weights see Figure S12 and Table S5.

The fact that the NN-weights (W4) performed better than the frequency-based weights (W3) indicates that the neural network learned more than the reaction frequency alone. To gain an intuition for the additional information the network could have learned, we built Escher maps of the citric acid cycle of *E. coli* and colored reactions by their mean recall over 500 iterations where we randomly deleted 30% of the reactions each time (Figure 6). Scheme W4 produced the highest recall (Figure 6D), followed by W3 (Figure 6C). We found that recall partially correlated with the reaction frequency in the training data for both W3 (Pearson r^2^ = 0.5, *p* = 1.06∗10^−116^) and W4 (Pearson r^2^ = 0.25, *p* = 1.22∗10^−26^, Figure S13) but that W4 also found some rare reactions that were more specific to *E. coli*, indicating that the network also learned specific co-occurrence patterns. The rest of the central metabolism of *E. coli* showed a similar pattern as the citric acid cycle (Figures S10 and S11).

### DNNGIOR-generated models have conservative but precise carbon usage profiles

**Figure 7:**
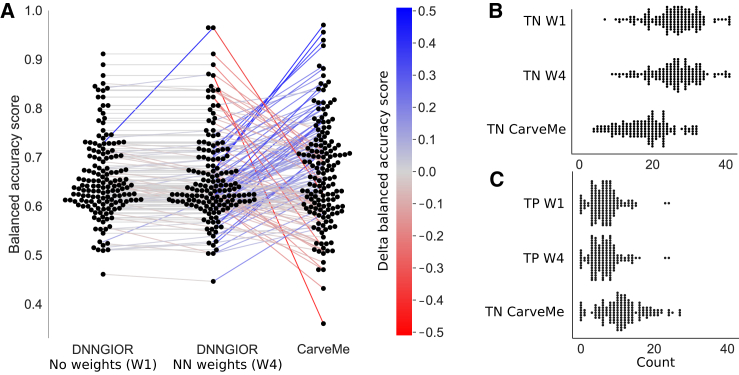
Accuracy of simulated carbon usage profiles (A–C) Swarm plots of the (A) Balanced accuracy score, (B) TNs, and (C) TPs of the carbon usage profiles of automatically constructed models compared to experimentally measured profiles. Lines connect scores belonging to the same models showing possible trends. (TPR = True Positive Rate. TNR = True Negative Rate, TP = True positives, TN = True negatives. Models that showed growth without any carbon source provided or did not grow on any of the carbon sources were omitted. The full carbon utilization profiles can be found in Table S6.

Finally, we provide an experimental benchmark by comparing the ability of models constructed with DNNGIOR and CarveMe^11^ to predict experimentally measured carbon usage profiles of 224 different bacteria.^34^ We found that although the balanced accuracy scores of DNNGIOR models were similar in range to the CarveMe models, some models performed worse and other models better with no significant trend either way. DNNGIOR’s half-interval algorithm tends to be more conservative in adding reactions than CarveMe’s algorithm, which resulted in more TNs but also fewer TPs (Figure 7B). Interesting to note are the balanced accuracy scores of W4 DNNGIOR models of Leaf412 and Leaf456, two *Methylophilus* species that showed high scores compared to both the W1 (+0,24) and CarveMe models (+0.34). These high scores are likely due to the low strain versatility, i.e., only glucose and methanol led to *in vitro* growth, which made an NN-guided accurate inclusion particularly effective.

In contrast to the internal validation, using NN-weights (W4) did not greatly improve accuracy compared to the gap-filling with no weights (W1). The similarity in scores illustrates that matching phenotypes remains challenging, and manual curation will remain valuable to resolve ambiguities.^34^ However, DNNGIOR weights provide additional information useful for both manual and automated model reconstruction efforts that represent the evolutionary associations between metabolic reactions throughout the bacterial domain. Interestingly, we observed that DNNGIOR performed better on larger than on smaller pathways, as we found a weak correlation between pathway size and F1-score (Pearson r^2^ = 0.23, *p* = 3.6∗10–7, Figure S14). This could reflect a stronger co-occurrence signal between reactions in larger than in smaller pathways.

## Discussion

### Future potential of the neural networks

The DNNGIOR neural network-based reaction weights significantly improved the gap-filling of GSMMs for a wide variety of bacteria. This success derives from the network learning aspects including the frequency and co-occurrence relationships between reactions. Additional features outside the scope of this study, such as reaction fluxes, pathway annotations, or environmental factors that were previously shown to be useful for gap-filling^26^^,^^27^^,^^28^^,^^37^^,^^38^^,^^39^ could also be incorporated, which could improve the prediction accuracy of the weights and subsequent gap-filling even further. Another promising avenue is using the network structure of the input data, including phylogenetic and metabolic networks. Graph convolution takes networks as input and may be a powerful tool to address such complex problems.^40^ A promising approach using hypergraph link prediction based on the stoichiometric matrix has recently been suggested as an alternative method for gap-filling metabolic networks, showing the potential of including the graph topology into the neural network.^27^ Combining this with the broader taxonomic signal found in our more diverse dataset could, in the future, improve performance across a broad range of organisms.

### Extensions to increase applicability

Currently, efforts are being made to reconcile the different reaction databases into MetaNetX.^41^ Once finished, a new neural network could be created that would use the reconciled database that would be more broadly applicable. In this paper we focused on ModelSEED models, but we have also created a version trained on CarveMe models showing similar results (Figure S15). This shows that it is possible to create neural networks for weighing the reactions in models from different sources. The neural network gives a prediction score for all reactions, not just for the missing ones. Currently, most of these predictions are ignored as our foucs was to fill in the missing reactions and retain the ones that were based on genome annotation, i.e., that have genetic evidence. However, mistakes during metagenome binning^14^^,^^15^ and annotation result not only in missing reactions (incomplete MAGs) but also reactions that are falsely attributed to a genome (contaminated MAGs) and thus spurious reactions in the corresponding metabolic models.^42^ Combining evidence of the predicted taxonomic affiliation of metagenomic contigs^43^ with the weights predicted by the DNNGIOR neural network could potentially help identify such erroneous reactions which could then be removed during model curation.

### Final remarks

We developed DNNGIOR, a neural network that predicts which metabolic reactions are present in a given bacterial strain, based on incomplete information. As the neural network learns about these reactions from known bacteria, it is particularly effective for scoring reactions that are relatively common, and for organisms that are relatively closely related to those present in the training dataset. Advanced users can tailor the training dataset to their specific needs, e.g., training models for certain biomes or taxa. The predicted weights can be used during gap-filling to improve the accuracy and overall quality of the reconstructed metabolic models. Increasingly, GSMMs are being used to interpret microbial metabolic traits, growth, or environmental associations. DNNGIOR should be a valuable tool to enhance the potential of these models.

## Resource availability

### Lead contact

Further inquiries and requests should be directed to and will be fulfilled by the lead contact: M.D. Boer (meineboer@gmail.com).

### Materials availability

No physical material was produced during this study.

### Data and code availability

• All relevant code is publicly available on GitHub (https://github.com/MGXlab/DNNGIOR) or as a pip package (https://pypi.org/project/dnngior/).
• All relevant data are freely available and found in supplementary data (Table S6. Carbon usage profiles of DNNGIOR (W1), DNNGIOR (W4), CarveMe and experimental, related to Figure 7. Growth of each strain (rows) has been scored on 45 carbon sources (columns). 1 = growth, 0 = no growth, NS = some residual growth cannot be excluded (experimental), NA = growth indetermined, Table S7. Minimal media and carbon sources, related to Star Methods and Figure 7. Composition of the medium used for simulation to determine the utilisation of different carbon sources by generated models, Table S8. Best per species dataset, related to Star Methods. Dataset of 11,700 best per species genomes (high completeness, low contamination), used as training dataset, Table S9. Best per genus dataset, related to Star Methods. Dataset of 1,659 best per genus genomes (high completeness, low contamination), used as testing dataset, Table S10. Database of BiGG reactions, related to Star Methods and Supplementary Figure S15. Database of all possible gap-filling candidates, with information on stoichiometry, origin, and aliases, Table S11. Database of ModelSEED reactions, related to related to Star Methods and Figure 4. Database of all possible gap-filling candidates, with information on stoichiometry, origin, reversibility and aliases), all other related data can be requested from the lead contact: M.D. Boer (meineboer@gmail.com).
• All other items are available upon request from the lead contact.

### Limitations of the study

The study still leaves room for improvement. First, the current study does not incorporate additional features such as reaction fluxes, pathway annotation, or environmental factors. Second, the network structure of the input data, such as phylogenetic and metabolic networks, is not utilized in the neural network. Finally, the neural network performance is linked to the training dataset, which may limit its effectiveness for more distantly related or uncommon organisms.

## Acknowledgments

The authors would like to extend their gratitude to the TBB group for moral support and to J.K. van Amerongen for technical support. Funding support for this work came from the UU-NIOZ project “Turning the tide” (NZ4543.26) awarded to B.E.D. and A.F.H., the European Research Council (ERC) Consolidator grant 865694: DiversiPHI, the 10.13039/501100001659Deutsche Forschungsgemeinschaft (DFG, German Research Foundation) under Germany’s Excellence Strategy—EXC 2051—Project-ID 390713860, and the Alexander von Humboldt Foundation in the context of an Alexander von Humboldt-Professorship founded by 10.13039/501100002347German Federal Ministry of Education and Research. C.M. acknowledges support by MiCRop Consortium (NWO/OCW grant no. 024.004.014).

## Author contributions

Writing—original draft, M.D.B.; writing review and editing, C.M., A.F.H., D.G., and B.E.D.; visualization, M.D.B.; software, M.D.B., H.Z., and D.G.; funding acquisition, A.F.H. and B.E.D.; supervision, C.M., A.F.H., D.G., and B.E.D.

## Declaration of interests

The authors declare no competing interests.

## STAR★Methods

### Key resources table

**Table:** 

REAGENT or RESOURCE	SOURCE	IDENTIFIER
**Software and algorithms**		

Tensorflow	Abadi et al.^39^	https://www.tensorflow.org/
COBRApy	Ebrahim et al.^7^	https://opencobra.github.io/cobrapy/
ModelSEEDpy	Henry et al.^9^	https://github.com/ModelSEED/ModelSEEDpy
Gurobi	Gurobi Optimization LLC.^44^	https://pypi.org/project/gurobipy/
CarveMe	Machado et al.^11^	https://carveme.readthedocs.io/en/latest/
Escher	King et al.^45^	https://escher.github.io/
SciPy	Virtanen et al.^46^	https://scipy.org/
DNNGIOR	This paper	https://github.com/MGXlab/DNNGIOR

### Method details

#### Collection and processing of the training and testing datasets

We constructed the training and testing datasets using genomes collected from the BV-BRC database^35^ (formerly PATRIC, accessed 26th April 2022). For training, one genome per species was selected based on sequencing quality scores using the formula: completeness - (5 ∗ contamination). Ties for this score were resolved by selecting the genome with the highest coarse consistency, a value provided by bv-brc that evaluates the functional completeness of a genome, as assessed by evalCon.^47^ This selection resulted in a dataset of 13,359 genomes (Table S8) that comprehensively represents the bacterial domain while reducing the risk of overfitting on well-studied species. From this dataset we selected one best genome from each of the 1,659 genera in the best-per-species dataset based on the same score, resulting in a training dataset of 11,700 genomes and a testing dataset of 1,659 genomes (Table S9). This ensured that the testing dataset contained diverse bacterial genomes, not biassed toward genera with more species that were different from those in the training dataset.

For the 1,659 genomes in the testing dataset, we created a phylogenetic tree using concatenated alignment of hits to HMM profiles of 71 single-copy marker genes that was used for visualizing and further investigating the performance of our approach. Phylogenetic distances in the tree represent the number of amino acid substitutions per site.

From all genomes, metabolic models were constructed using either either ModelSEED^9^ or CarveMe^11^ and the set of gene-associated reactions was determined. From these models we determined the total set of reactions that were annotated in the 13,359 genomes, resulting in a “pan-reactome” of the bacterial domain within the ModelSEED and BiGG databases (*n* = 2543 and 4240 reactions, respectively). These pan-reactomes contain all reactions for which predictions can be made, this represents a majority of gene-associated bacterial reactions (Figure S16). Most of the other reactions in the ModelSEED and BiGG databases either originate from non-bacterial organisms (e.g., plants, fungi, animals) or are artificial reactions. Both these categories of reactions are not associated with bacterial genes, and therefore do not appear in the draft models or associated reactome. We decided against including non-gene-associated reactions in the training data to avoid learning the biases that automated tools introduce when including non-gene associated reactions. For every model in the training and testing datasets, we constructed a binary array describing which reactions were present, resulting in an incidence matrix of all reactions in all genomes.

During training we repeatedly deleted 30% ($\underline{n}\approx 300$) of the reactions in each genome, this was done 30 times for each of the 10,700 genomes resulting in a training dataset of 351,000 incomplete reaction sets for optimal performance (Figure S17). Reactions were randomly deleted either according to a uniform probability (Equation 1a) or with a bias toward lower frequency reactions (Equation 1b). We used the uniform deletion probability for all of the analyses except for the analysis of the effect of reaction deletion bias.

Probability function for uniform (a) or weighted (b) deletion.

Equation 1a $$a)\;D_{w}\left({frac}_{r}\right)=0.3$$

Equation 1b $$b)\;D_{w}\left({frac}_{r}\right)=1-\frac{1}{1+e^{10*\left({frac}_{r}-0.5\right)}}$$

We also generated several additional training datasets with certain phyla purposefully excluded to explore the importance of a full representation of the bacterial domain. For each genome, the original reaction sets as predicted by the genome-based draft reconstructions were used as truth. Testing datasets were created in a similar manner.

Finally we also collected models from the AGORA2 collection^22^ as well as their associated genomes from the bv-brc database^35^ for evaluation of prediction and gap-filling accuracy against 7,302 (semi-)curated models.

#### Hyper-parameterization and loss function of the neural network

Two neural networks were built, one for predicting ModelSEED reactions and one for predicting CarveMe or BiGG reactions. Both neural networks were built using Tensorflow^48^ v2.0.0. Their topology consists of an input and output layer of 2,453 or 4,240 nodes (one for each reaction in the ModelSEED or CarveMe pan-reactomes respectively) and three hidden layers. All layers were fully connected resulting in a network with 1,260,697 or 2,306,960 parameters respectively. The optimizer used for training both networks was the Adam optimizer^45^ with the following parameters: learning rate = 0.005, beta1 = 0.9, beta2 = 0.999, epsilon = 1.0e-8, decay = 0.01. Hyper-parameterization was performed in 100 trials using the Optuna package^49^ v2.0, resulting in the following hyper-parameters: number of nodes per hidden layer = 256, batch size = 50, number of hidden layers = 3, dropout = 0.1, number of epochs = 10.

For the loss function, we used a customized version of the binary cross-entropy function (CE, Equation 2).

Loss function based on binary cross-entropy^50^ to calculate the difference between the neural network output ($\vec{O}$) and the correct reactions ($\vec{T}$). $\vec{I}$ is the input vector of the NN, b_0_ is the absent class scaling factor.

Equation 2 $$m\vec{CE}=\left(\vec{1}-\vec{I}\right)\circ \left[\left(1-b_{0}\right)\;\left\{\vec{\;T}\circ \log\;\left(\vec{O}\right)\;\right\}-b_{0}\left\{\left(\vec{1}-\vec{T}\right)\circ \log\;\left(\vec{1}-\vec{O}\right)\;\right\}\right]$$

CE is calculated as the log-loss of the difference between what the network predicts ($\vec{O}$) and the truth ($\vec{T}$). We introduced two adaptations to increase the performance based on our training data. First, as for a given reaction set only ∼960 of the possible 2,457 reactions are present, we introduced a scaling factor ($b_{0}=0.3$) that allowed us to scale the loss of the two classes (absent and present). We multiplied $b_{0}$ by the loss for the absent class and $1-b_{0}$ by the loss for the present class. Second, we added a masking vector (1-T) that allowed us to exclude the loss associated with predictions for reactions that were already known to be part of the genome, i.e., those given as input to the neural network. We multiplied the loss of both classes element-wise b $\vec{1}-\vec{I}$ where $\vec{I}$ is the vector of reactions given as input. This adaptation ensures that the neural network learns to complete the reaction set and does not simply repeat the input.

#### Gap-filling algorithm and database

After predicting weights for all reactions, we used those weights to guide a half-interval search for the minimal set of reactions that simultaneously has a high probability and generates biomass flux that is greater than zero. The half-interval gap-filling algorithm was adapted from Latendresse.^25^ Briefly, this algorithm iteratively minimizes the following objective function with linear programming conditional on flux through the biomass reaction (f_b_):

Objective function of the half-interval gap-filling algorithm that optimizes gap-filling of an incomplete metabolic network based on weights and flux of reactions in a network.

Equation 3 $$\sum_{r\in m}c_{r}f_{r}\;|\;f_{b}>0$$

This sums over all reactions (r) in the candidate set (M), with f_r_ the flux through reaction r and c_r_ a user-defined cost used to implement the different weighting schemes during gap-filling. By using linear programming, the runtime is reduced by up to two orders of magnitude compared to mixed integer linear programming.^25^ The objective was solved using the gurobipy package.^44^

For ModelSEED models the default ‘bio1’ reaction was used as biomass reaction and for BiGG the CarveMe ‘Growth’ reaction was used. The reaction database from where reactions were selected was downloaded from the BiGG website (http://BiGG.ucsd.edu, Table S10) for the BiGG and CarveMe models and from the ModelSEED website (https://modelseed.org, Table S11) for the ModelSEED models. From these databases, biomass reactions were removed as they are generally artificial, added before gap-filling and not predicted by the DNNGIOR Neural Network. Reversible reactions were split into two reactions, one for each direction. The algorithm can also take into account different media compositions, as these may affect the solution.

#### Curated genome-scale metabolic models

We selected six high-quality manually curated models based on literature (iML1515,^17^ iJB785,^51^ iYL1228,^52^ iND750,^53^ iYO844,^54^ and iYS854^55^ from the BiGG database.^56^ We deleted in 10x replicate 30% of reactions from these models and gap-filled them with the same four weighting schemes as for gap-filling the draft-models (see section above). For these models the neural network trained on CarveMe models was used as the reaction identifiers matched those from the curated models. To illustrate which reactions are most likely to be found with the different weighting schemes we also deleted in 500x replicate 30% of reactions from the *Escherichia coli* model (iML1515) and constructed an Escher^57^ map of the central metabolism. For the test where only essential reactions were removed, the find_essential_reactions function was used from COBRApy^7^ (v0.28.0).

### Quantification and statistical analysis

#### Weighting schemes for guiding the gap-filling algorithm

Four weighting schemes were used to guide the half-interval algorithm, namely: W1. No weights, W2. Naive binary weights, W3. Frequency weights and W4. NN weights (see Equation 4)**.** For W1, all reactions in the database (*n* = 43,775) are given the default cost (c_r_ = 50). For W2, a low fixed cost (c_r_ = 1) is given to reactions that are present at least once in the training dataset (*R*_*train*_, *n* = 2457), and the default cost to all other reactions in the database. For W3 a lower cost is given to a reaction if it is present in a higher fraction of genomes in the training dataset. For W4, a lower cost is given to reactions that have a higher prediction score. Because the half-interval gap-filling algorithm tries to minimize the product of a reaction’s cost and flux, reactions that are given lower costs are given more weight in the final solution.

Four weighting schemes to guide the half-interval gap-filling algorithm, see text for details.

Equation 4 $$\begin{array}{c} W1=50\\ W2=\{1,r\in R_{train};\;50,r\notin R_{train}\}\\ \begin{array}{c} W3=1-{\text{frac}}_{r}\\ W4=1-p_{NN} \end{array} \end{array}$$

We compared the four different weighting schemes by gap-filling the models that were constructed based on the 1,659 genomes from the testing dataset from which 30% of reactions ($\underline{n}\approx 300$) was deleted at random in triplicate. After gap-filling, we counted which removed reactions were re-added correctly (TPs) or not (FNs), and which reactions were falsely added (FPs). These were used to calculate an F1-score for the different weighting schemes.

#### Validation of gap-filled models based on experimental data

We obtained the genome sequences of 224 bacterial isolates from Arabidopsis thaliana leaves.^34^ Draft models were constructed using ModelSEEDpy^58^ v0.3.0 and gap-filled in a minimal medium (Table S7) with and without DNNGIOR neural network weights. CarveMe^11^ models were built using version v1.6.0 with default parameters and the M9 minimal medium provided by the package. Simulated carbon utilization profiles were established by measuring flux through the biomass function (‘bio1' for DNNGIOR models and ‘Growth’ for CarveMe models) on 45 different carbon sources using cobrapy^7^ v0.28.0. Balanced accuracy scores were calculated by comparing the simulated carbon utilization profiles to those that were measured *in vitro*^34^ using Equation 5.

Balanced accuracy score.

Equation 5 Balancedaccuracy=(specificity+recall)/2

#### Statistical tests

Statistical significance of the correlations between prediction accuracy and reactions frequency, phylogenetic distance or KEGG pathway size was determined using the pearsonr function from SciPy^46^ with default parameters. Determining the significance of the difference in mean F1-score between including and excluding phyla or the different weighting schemes was performed using the Wilcoxon rank test from SciPy^46^ using default parameters.

## Supplemental information

Document S1. Figures S1–S18 and Tables S1–S5

Table S6. Carbon usage profiles of DNNGIOR (W1), DNNGIOR (W4), CarveMe and experimental, related to Figure 7. Growth of each strain (rows) has been scored on 45 carbon sources (columns). 1 = growth, 0 = no growth, NS = some residual growth cannot be excluded (experimental), NA = growth indetermined

Table S7. Minimal media and carbon sources, related to Star Methods and Figure 7. Composition of the medium used for simulation to determine the utilisation of different carbon sources by generated models

Table S8. Best per species dataset, related to Star Methods. Dataset of 11,700 best per species genomes (high completeness, low contamination), used as training dataset

Table S9. Best per genus dataset, related to Star Methods. Dataset of 1,659 best per genus genomes (high completeness, low contamination), used as testing dataset

Table S10. Database of BiGG reactions, related to Star Methods and Supplementary Figure S15. Database of all possible gap-filling candidates, with information on stoichiometry, origin, and aliases

Table S11. Database of ModelSEED reactions, related to related to Star Methods and Figure 4. Database of all possible gap-filling candidates, with information on stoichiometry, origin, reversibility and aliases
