# Supplementary material for: Improving genome-scale metabolic models of incomplete genomes with deep learning
Source: iScience. 2024 Nov 7;27(12):111349. doi: 10.1016/j.isci.2024.111349 (PMC11629236; doi:10.1016/j.isci.2024.111349)
Supplement: Document S1. Figures S1–S18 and Tables S1–S5 [file mmc1.pdf]

iScience, Volume *27*

Supplemental information

Improving genome-scale metabolic models of incomplete genomes with deep learning

Meine D. Boer, Chrats Melkonian, Haris Zafeiropoulos, Andreas F. Haas, Daniel R. Garza, and Bas E. Dutilh

Supplementary Figures

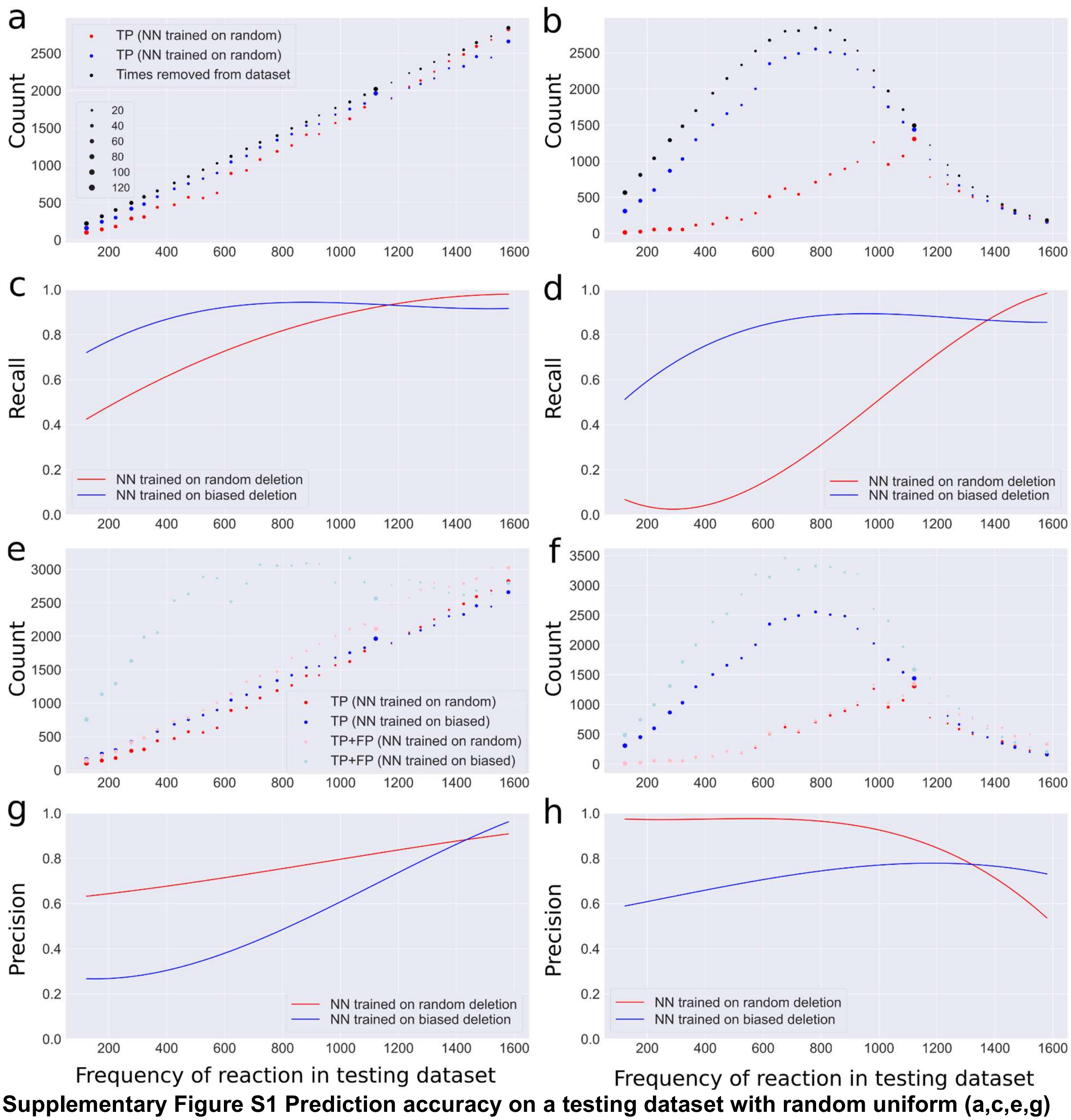

Supplementary Figure S1 Prediction accuracy on a testing dataset with random uniform (a,c,e,g) and biassed deletion (b,d,f,h), related to Figure 2. TP = True Positives, FP = False Positives, FN = False Negatives, Recall = TP/(TP+FN), Precision = TP/(TP+FP), NN = Neural Network. a-b) TP counts for both the NN trained on data with random deletion and the NN trained on the training data with biassed deletion and the count for total number of times reactions are removed (TP+FN) for a testing dataset with a) random uniform and b) biassed deletion (see methods). The reactions are averaged over frequency bins of 50 genomes. The dot size corresponds to the number of reactions in the bin. c-d) Polynomial regression curves for the recall of both NNs for a testing dataset with c) random uniform and d) biassed deletion. e-f) Counts of TPs and (TP + FP) for both NNs for a testing dataset with e) random uniform and biassed deletion. Binning of reactions was done as in panel a-b. g-h) Polynomial regression curves of precision of both NNs for a testing dataset with g) random uniform and h) biassed deletion.

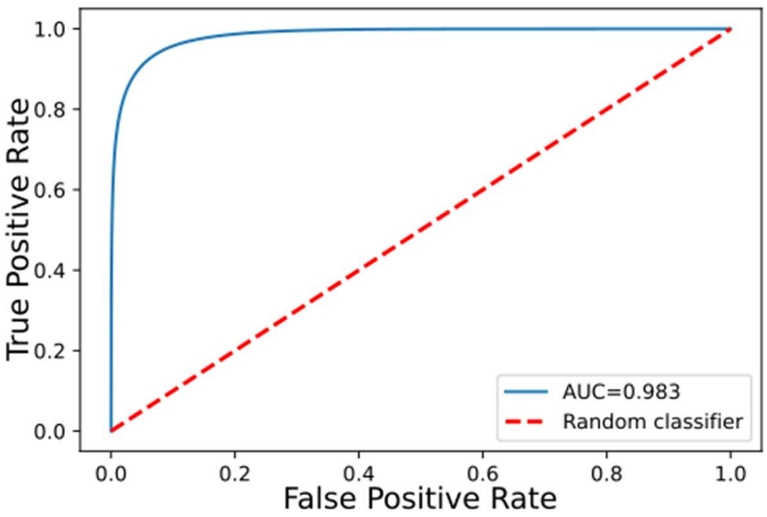

Supplementary Figure S2 ROC-curve, related to Figure 2: ROC plot of predictions made by the neural network on the one-per-genus testing dataset, AUC = area under the curve. The dotted line corresponds to a random classifier.

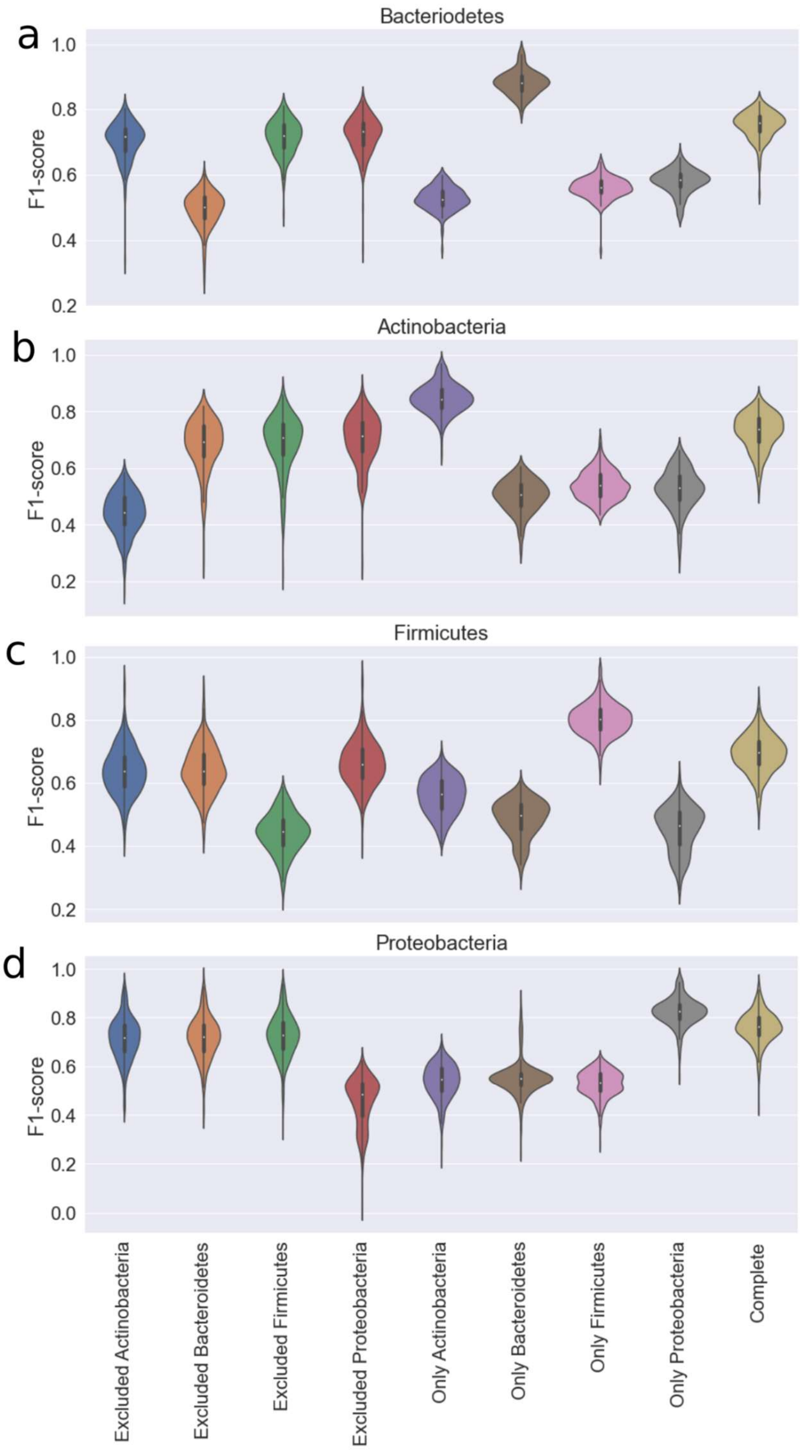

Supplementary Figure S3: Exclusive and singular inclusive training of different phyla, related to Figure 3. Violin plots of the prediction accuracy on different when a phylum is excluded from the training data or when the training data contains exclusively one Phylum, corrected for training set size for A) Bacteroidetes B) Actinobacteria C) Firmicutes and D) Proteobacteria. Interior of the violin shows the median and the interquartile range.

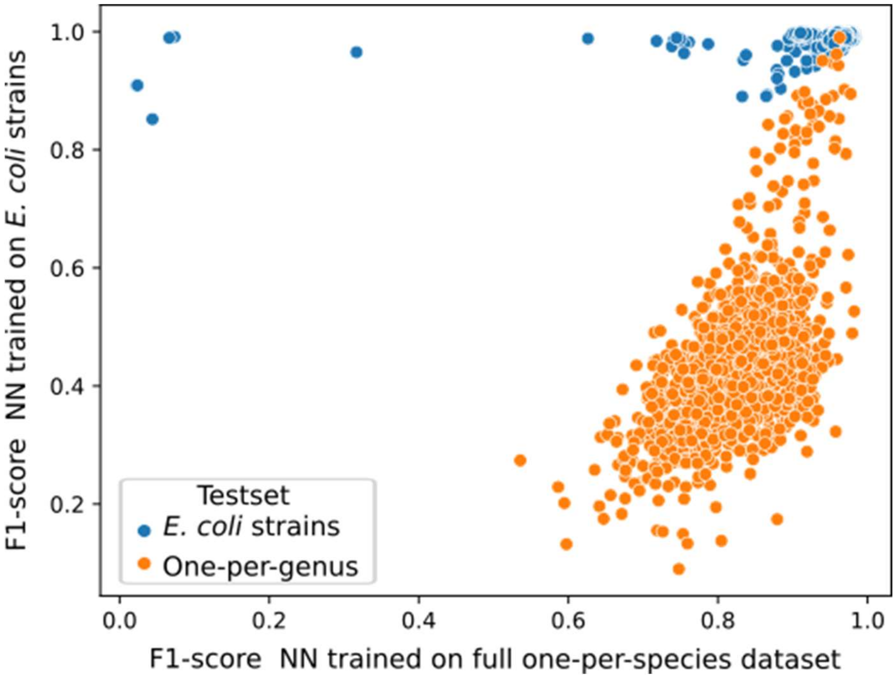

Supplementary Figure S4: **Specialised training comparison, related to Figure 3.** Comparison specialized versus general neural network. Prediction accuracy of a specialized (y-axis) and general (x-axis) neural network on the E. coli strains (blue) and One-per-genus (orange) testing datasets.

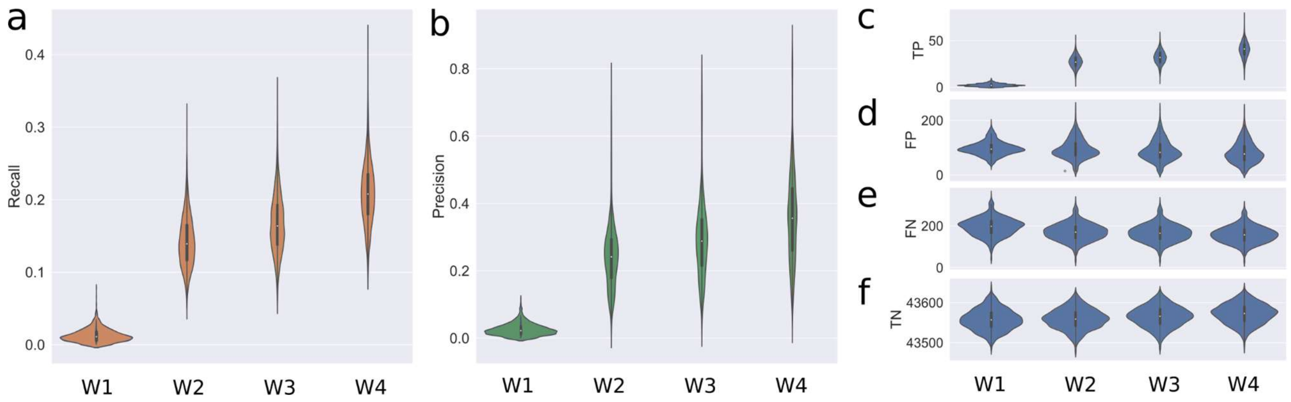

Supplementary Figure S5 Weighted gap-filling of draft models, related to Figure 4. Violin plots of a) Recall-scores, b) Precision scores, c) TPs, d) FPs, e) FNs and f) TNs of the gap-filling of 1,659 models from the testing dataset, from which we randomly deleted 30% of reactions in triplicate. These reduced models were gap-filled using four different weighting schemes (Equation 3). For W1 ("No weights") all reactions in the database are weighted equally. For W2 ("Naive binary weights") all reactions that are present in the training data were given the same weights. For W3 ("Frequency-based weights") the frequency of the reaction was used to weigh reactions. For W4 ("NN-weights") the prediction scores generated by the DNNGIOR neural network were used. Interior of the violin shows the median and the interquartile range.

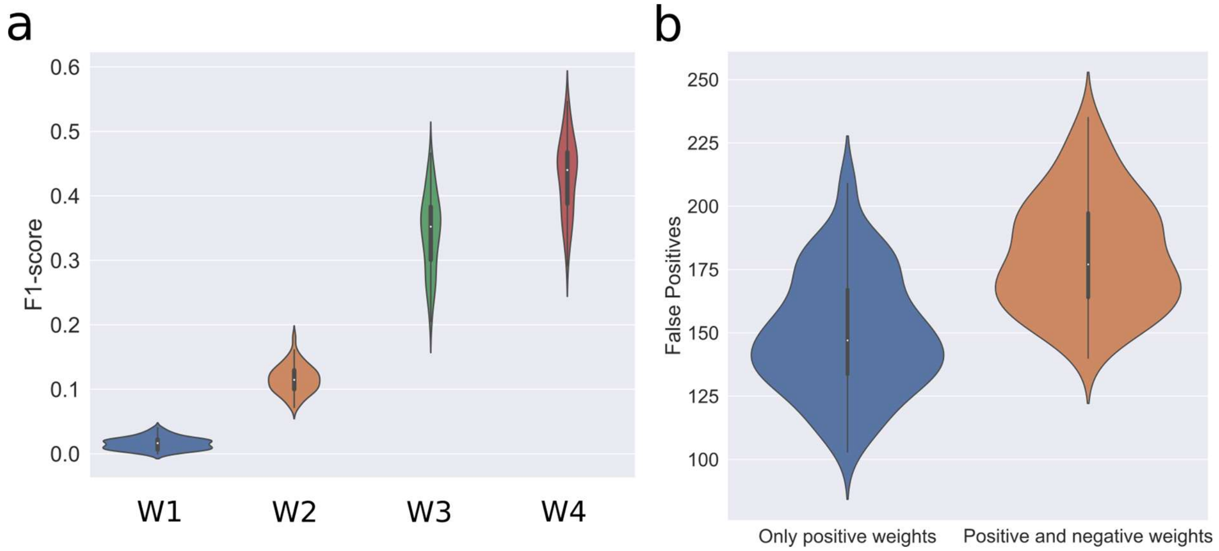

Supplementary Figure S6: Weighted gap-filling of draft models with negative weights, related to Figure 4. Violin plots of F1-scores of the gap-filling of 1,659 models in the testing dataset, from which we randomly deleted 30% of reactions in triplicate. These reduced models were gap-filled with four different weighting schemes where weights ranged from -1 to 1. For W1*: "Naive binary weights" all reactions that are present in the training set were given the same weight of 0. For W3*: "Frequency-based weights" the frequency of the reaction was used ($W_{freq} = 1 - 2 * R_{freq}$). For W4* "NN-weights" the prediction scores generated by the neural network were used ($W_{NN} = 1 - 2 * p_{NN}$). Interior of the violin shows the median and the interquartile range.

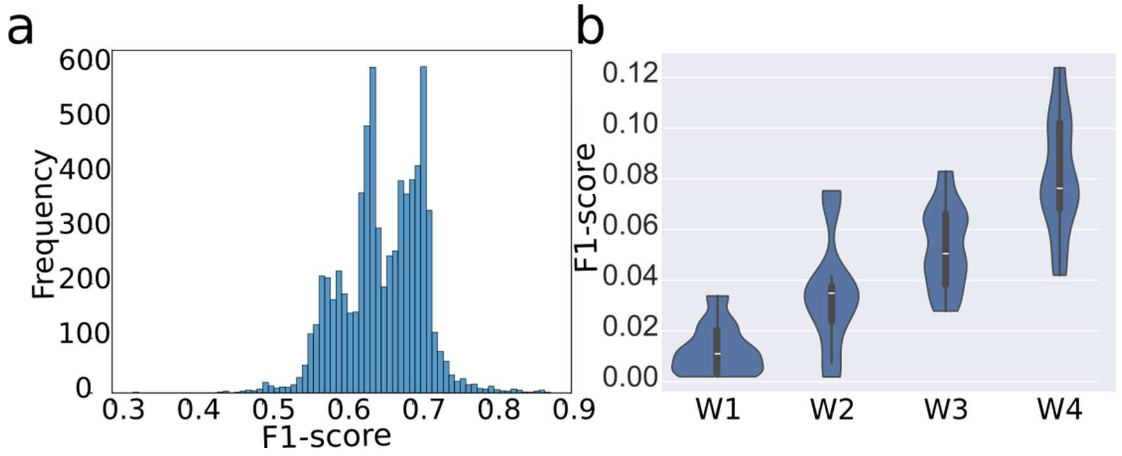

Supplementary Figure S7: Comparison with Agora2 models, related to Figure 4. a) Prediction accuracy of DNNGIOR predictions on the gene-associated reactions in the 7302 models from the AGORA2 collection (23) using the translation from vmh provided within the models b) Violin plots of the gap-filling accuracy of DNNGIOR constructed models using AGORA2 models as truth for different weighting schemes. For W1 ("No weights") all reactions in the database are weighted equally. For W2 ("Naive binary weights") all reactions that are present in the training data were given the same weights. For W3 ("Frequency-based weights") the frequency of the reaction was used to weigh reactions. For W4 ("NN-weights") the prediction scores generated by the DNNGIOR neural network were used. Presumably, the difference in absolute F1-score is due to (semi) automatically curated reactions added by DEMETER (29) that were not essential for growth, (see also Figure 5 and 6). Missing translations between vmh and ModelSEED identifiers could also have lowered this score. Interior of the violin shows the median and the interquartile range.

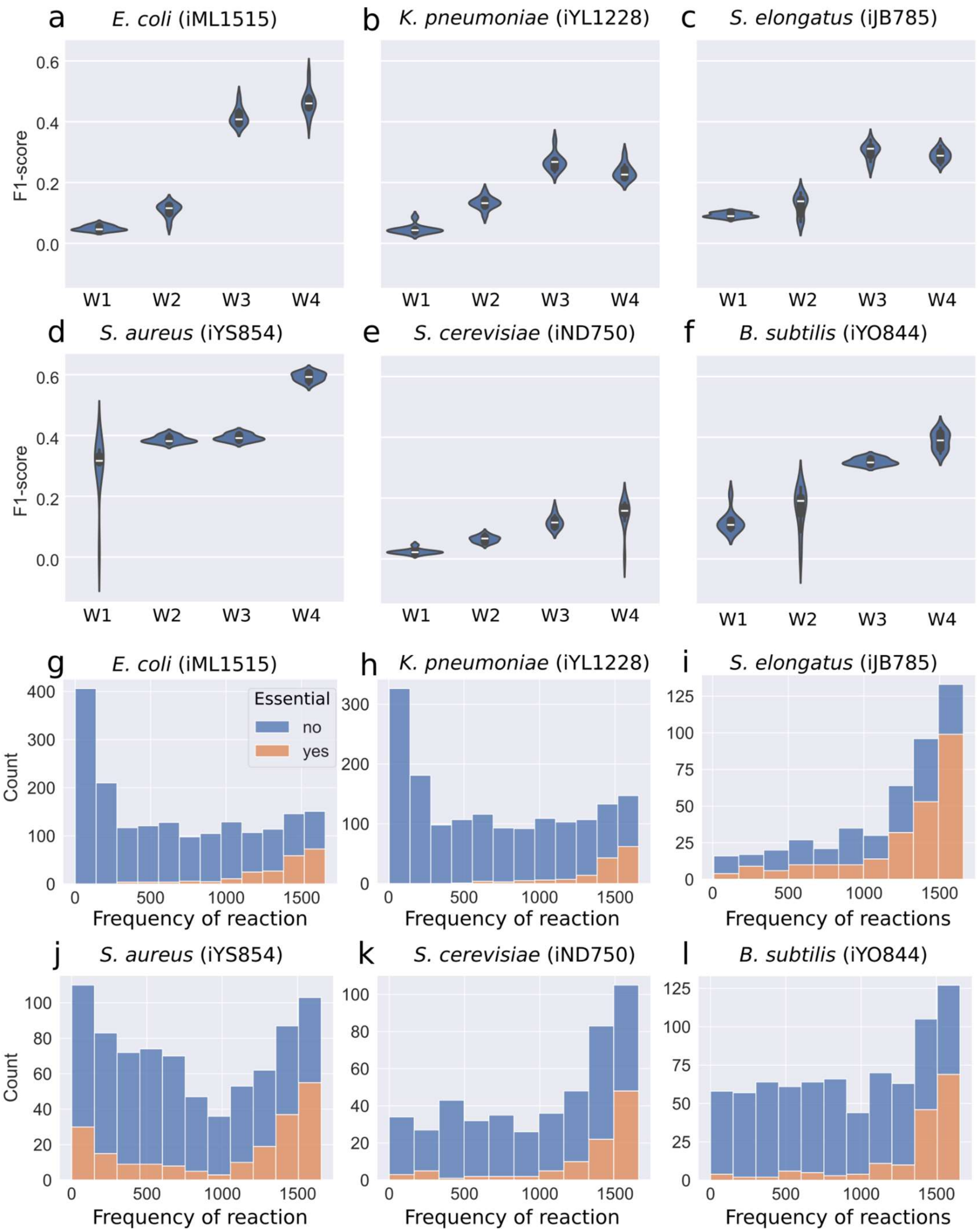

Supplementary Figure S8: **Essential reaction gap-filling, related to Figure 5**. Violin plots of F1-scores of the gap-filling of curated models: a) E. coli (iML1515), b) K. pneumoniae (iYL1228), c) S. elongatus (iJB785), d) S. aureus (iYS854), e) S. cerevisiae (iND750), and f) B. subtilis (iYO844). We randomly deleted 30% of reactions 10 times from the set of essential reactions as determined by Cobrapy (7). These reduced models were gap-filled using four different weighting schemes (Equation 3). For W1 ("No weights") all reactions in the database are weighted equally. For W2 ("Naive binary weights") all reactions that are present in the training data were given the same weights. For W3 ("Frequency-based weights") the frequency of the reaction was used to weigh reactions. For W4 ("NN-weights") the prediction scores generated by the DNNGIOR neural network were used. Interior of the violin shows the median and the interquartile range. Stacked histogram of frequency of reaction in the one-per-genus testing dataset, coloured by reaction essentiality for g) E. coli (iML1515), h) K. pneumoniae (iYL12278), i) S. elongatus (iJB785), j) S. aureus (iYS854), k) S. cerevisiae (iND750), l) B. subtilis (iYO844).

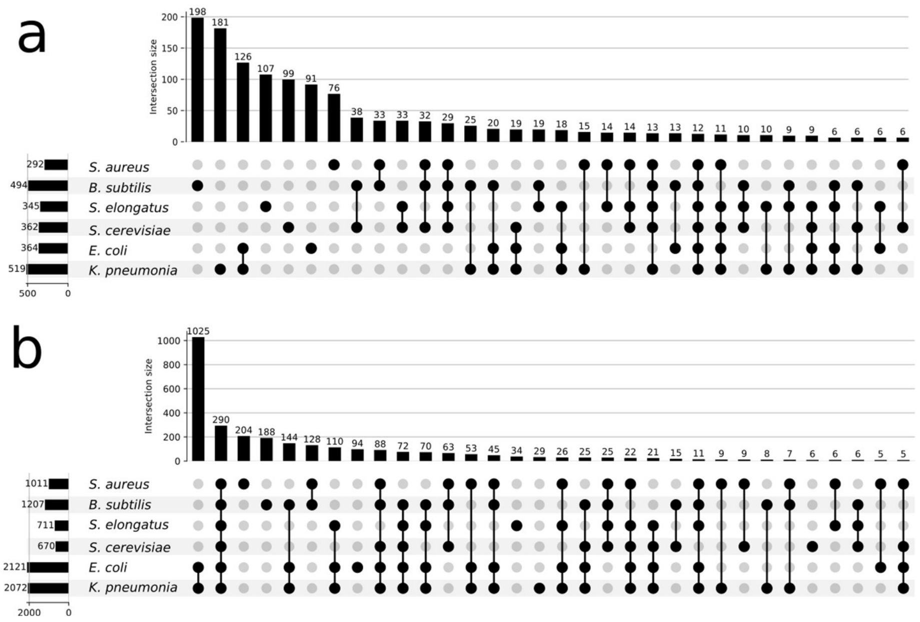

Supplementary Figure S9: DNNGIOR neural network overlap, related to Figure 6. Upset plots showing the overlap in: a) predicted missing reactions and b) all predictions made for 6 manually curated models, unions with size < 5 are omitted.

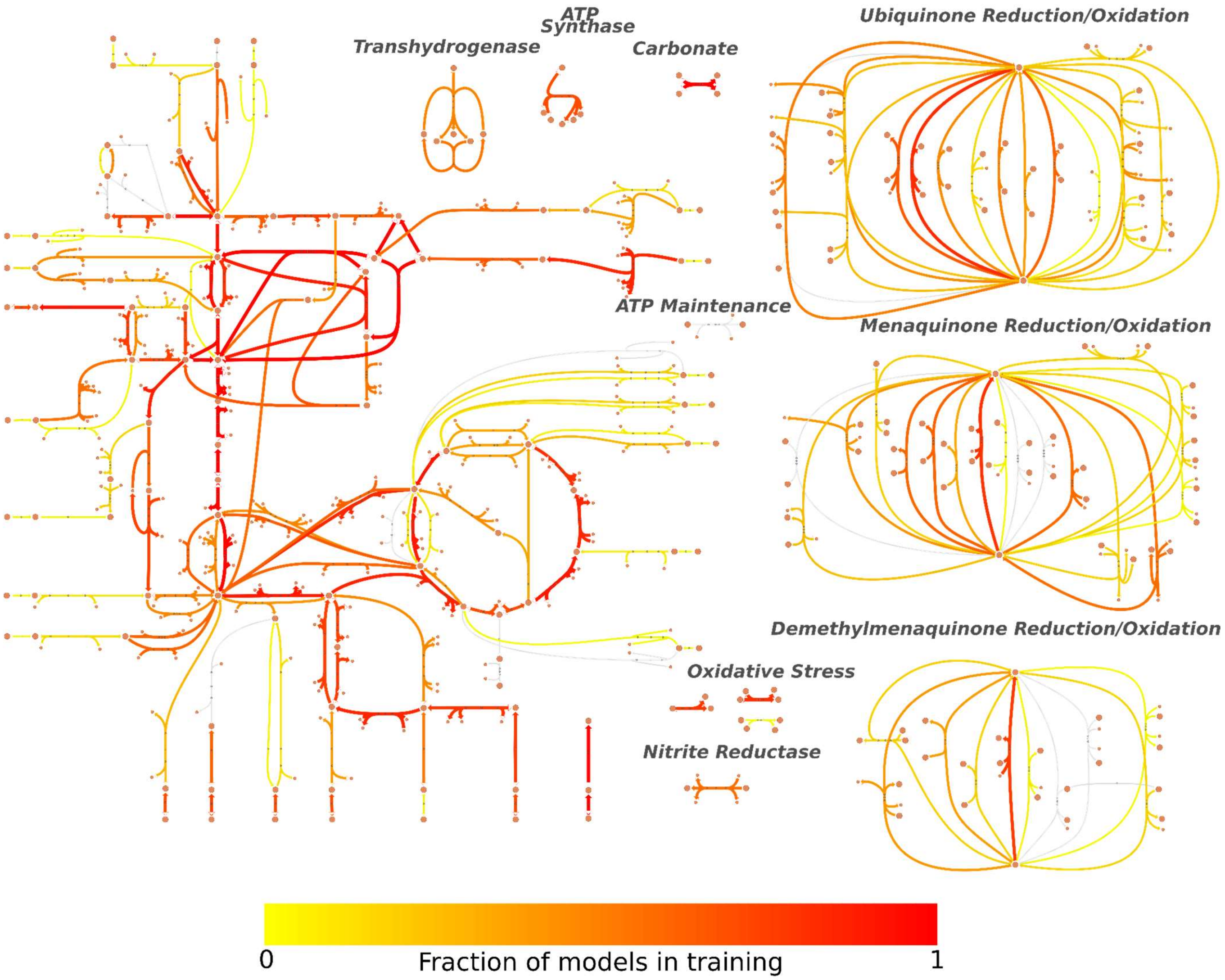

Supplementary Figure S10: Frequency of the reactions in the central metabolism of *E. coli,* related to Figure 6. Escher map of the central metabolism coloured by frequency of the reactions in the models of the training dataset. IDs for secondary metabolites were omitted, reactions in grey were absent from the training dataset.

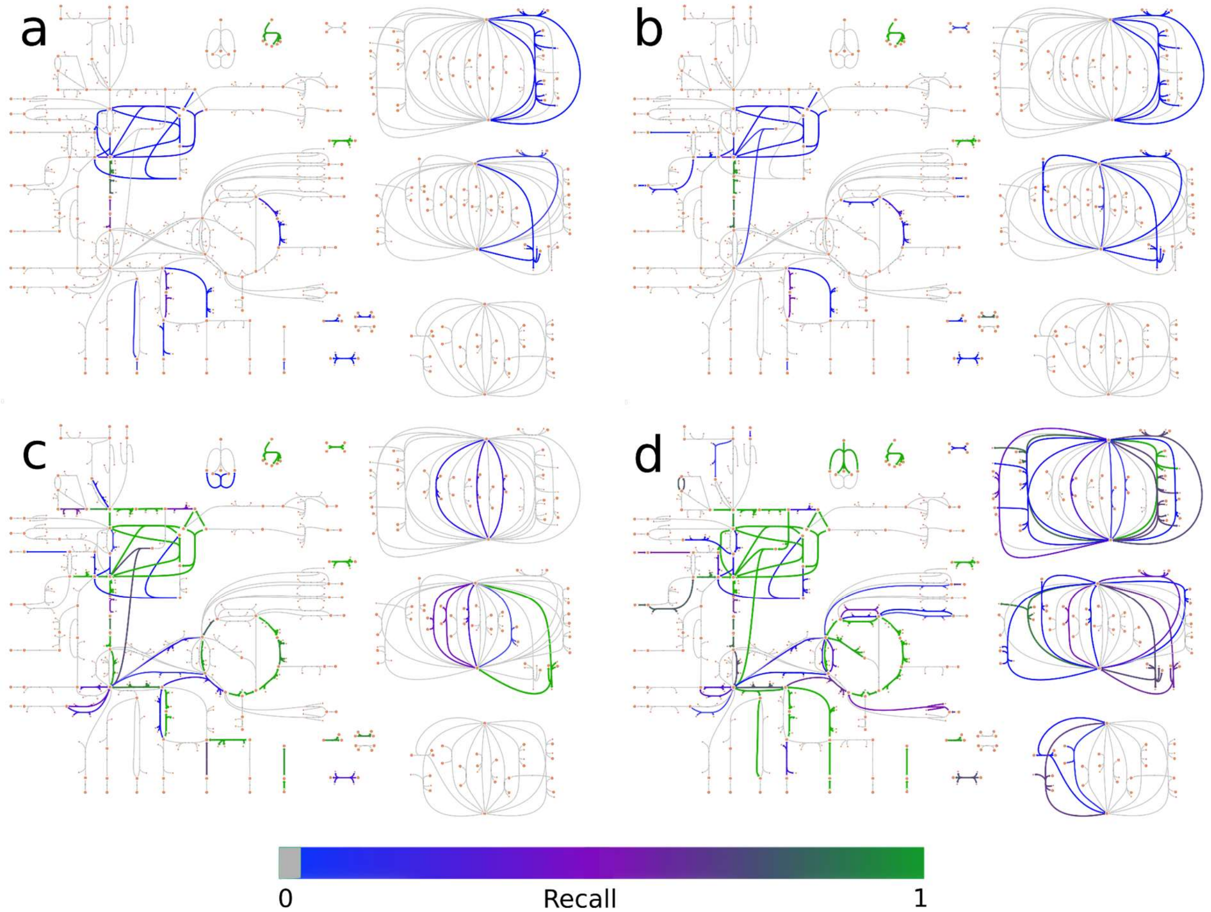

Supplementary Figure S11: Recall of the reactions in the central metabolism of *E. coli*, related to Figure 6. Escher maps of the central metabolism as in Supplementary Figure S6, coloured by the recall after gap-filling models, from which we randomly deleted 30% of the reactions 500 times. Reduced models were gap-filled using four different weighting schemes: a) W1: no weights b) W2: Naive binary weights c) W3: Frequency-based weights and d) W4: NN-weights. IDs for secondary metabolites were omitted.

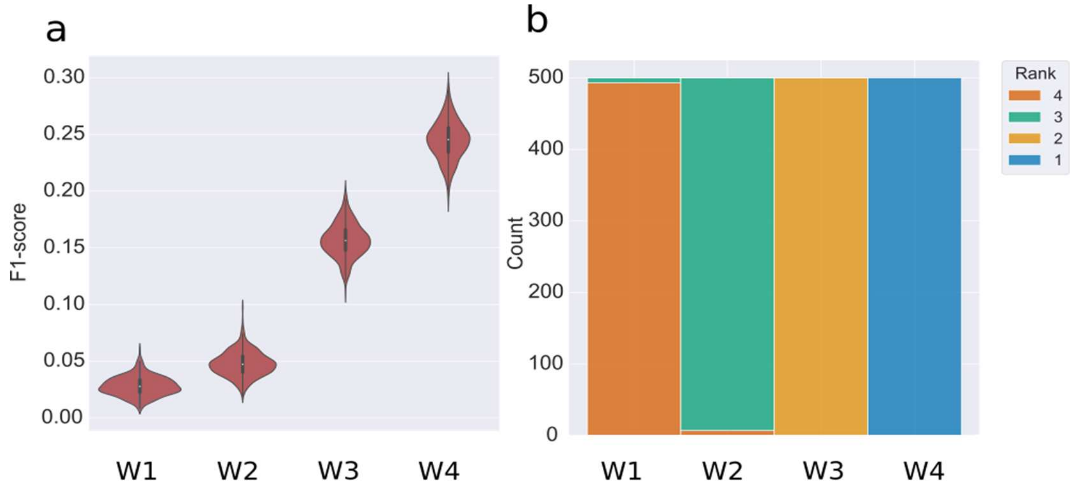

Supplementary Figure S12: Weighted gap-filling of iML1515 used for Escher mapping, related to Figure 6. a) Violin plot of F1-scores of the gap-filling of iML1515 (*E. coli*), from which we randomly deleted 30% of reactions 500 times. These reduced models were gap-filled using four different weighting schemes (Equation 3). For W1 ("No weights") all reactions in the database are weighted equally. For W2 ("Naive binary weights") all reactions that are present in the training data were given the same weights. For W3 ("Frequency-based weights") the frequency of the reaction was used to weigh reactions. For W4 ("NN-weights") the prediction scores generated by the DNNGIOR neural network were used. Interior of the violin shows the median and the interquartile range. b) Distribution of the ranks of the F1-scores for different weights with four being the worst ranking and one being the best.

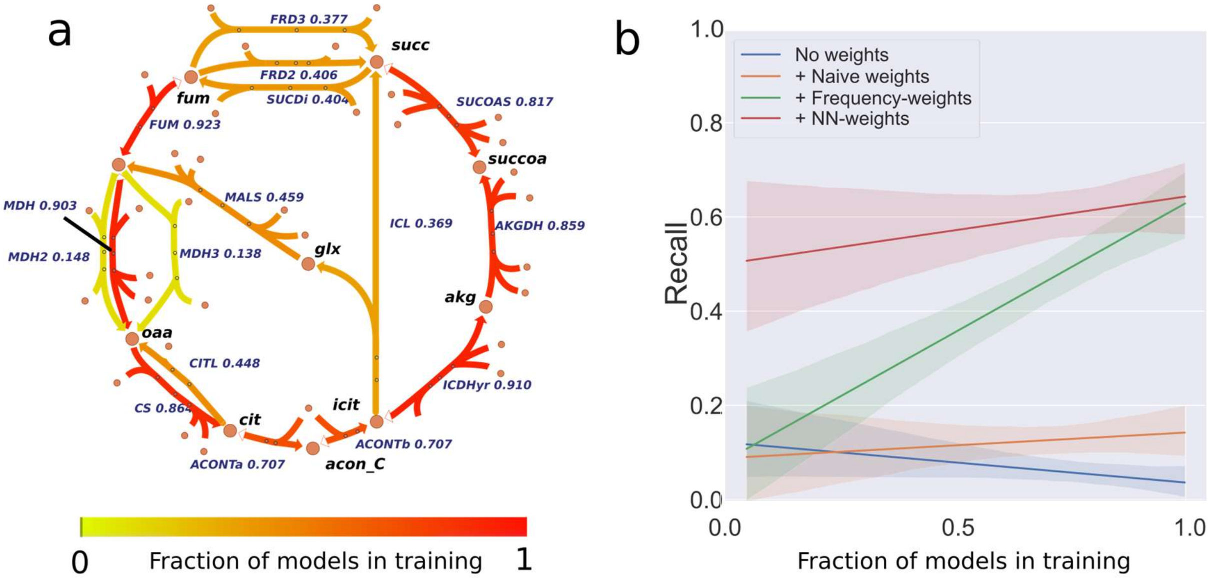

Supplementary Figure S13: Frequency and recall of reactions in the citric acid cycle, related to Figure 6. a) Escher map of the citric acid cycle coloured by frequency. IDs for secondary metabolites are omitted. b) Polynomial regression plots of recall plotted against the fraction of models in training that a reaction is present in for different weighting schemes. Lines show trends and 95% confidence intervals.

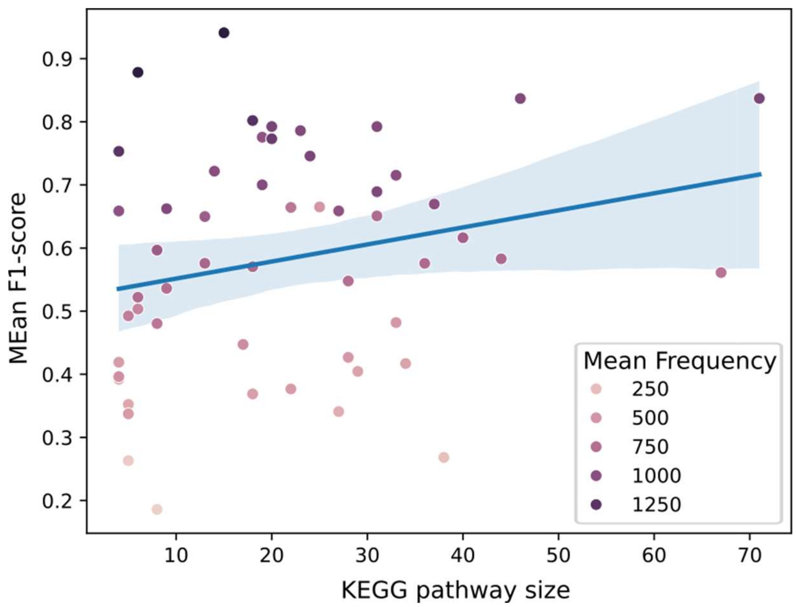

Supplementary Figure S14: **KEGG pathways prediction accuracy, related to Figure 7.** Scatterplot of mean F1-scores of NN-predictions in the one-per-genus testing dataset versus the number of reactions in KEGG pathways (57), coloured by the average frequency of the reactions in the one-per-genus testing dataset. Line shows trend and 95% confidence interval (Pearson r^2 = 0.23, p = 3.6*10-7).

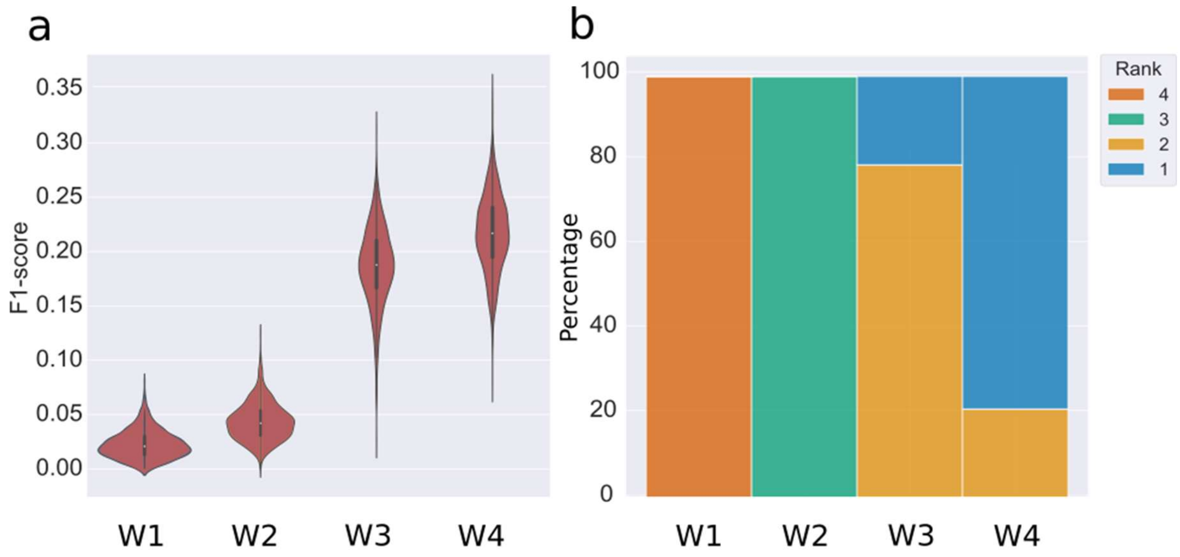

Supplementary Figure S15: **Weighted gap-filling of CarveMe models, related to Figure 4.** a) Violin plot of F1-scores of the gap-filling of 1,659 models from the testing dataset, from which we randomly deleted 30% of reactions in triplicate. These reduced models were gap-filled using four different weighting schemes (Equation 3). For W1 ("No weights") all reactions in the database are weighted equally. For W2 ("Naive binary weights") all reactions that are present in the training data were given the same weights. For W3 ("Frequency-based weights") the frequency of the reaction was used to weigh reactions. For W4 ("NN-weights") the prediction scores generated by the DNNGIOR neural network were used. Interior of the violin shows the median and the interquartile range. b) Distribution of the ranks of the F1-scores for different weights with four being the worst ranking and one being the best.

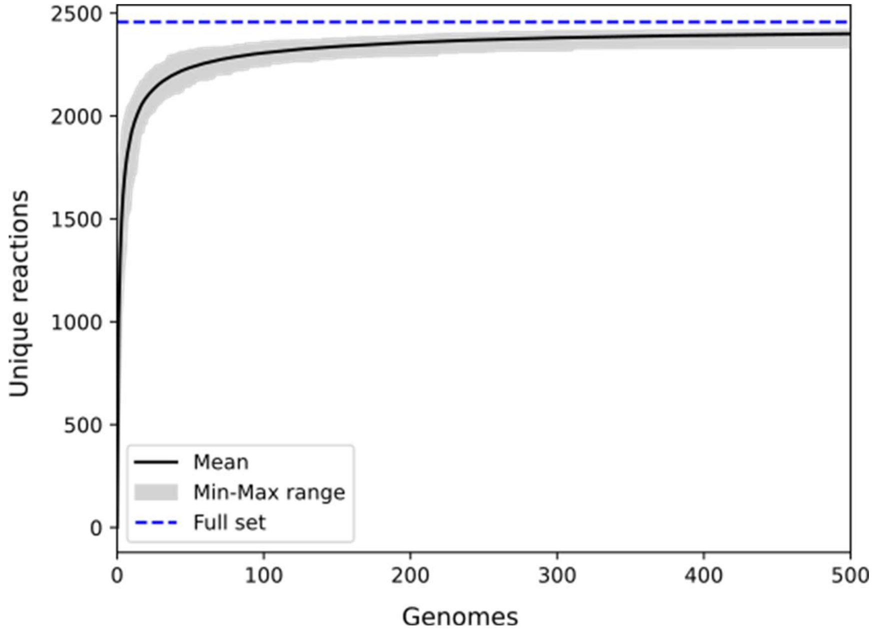

Supplementary Figure S16: Rarefaction curve of the reactome, related to Star Methods section "Collection and processing of the training and testing data". Simulated growth of reactome by sampling of 20 times 500 genomes from the training dataset. The dotted line corresponds to the reactome size of the full set of 13,359 genomes.

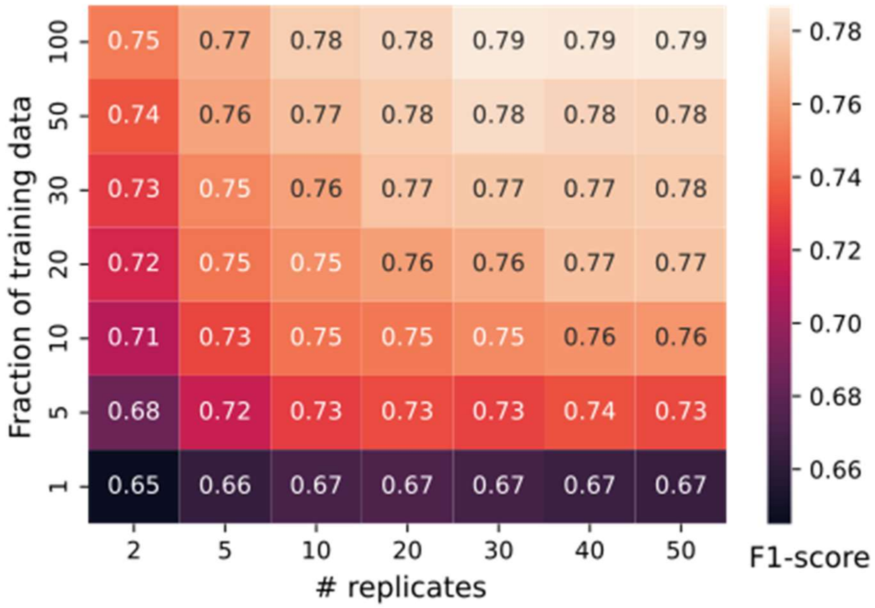

Supplementary Figure S17: Effect of size training data, related to Star Methods section "Collection of processing of training data". Heatmap of F1-scores of predictions by a neural network with different sizes of training set. Fraction of training data refers to the fraction of the total 13,359 genomes using for training, # replicates is the number of times 30% of reactions were removed to generate features.

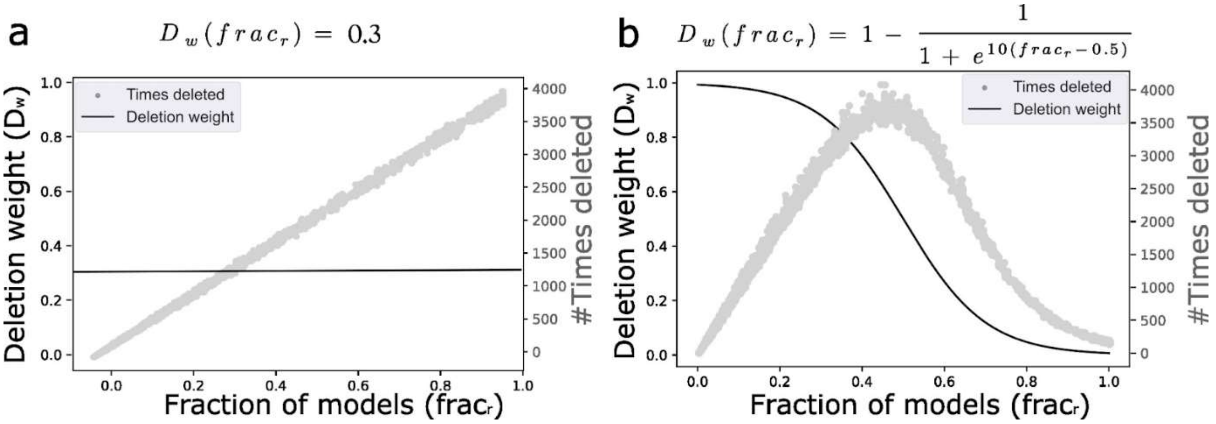

Supplementary Figure S18: **Reaction deletion probability distributions, related to Star Methods section: "Collection and processing of the training and testing data".** Probability function for uniform (a) or weighted (b) deletion of reactions in the training and testing datasets. Based on these weights, the scatter plots show how often reactions were actually deleted on average per replicate. ***$Frac_r$*** represents the fraction of genomes in the training dataset that include a reaction

Supplementary Tables

Supplementary Table S1: NN-predictions scores metrics, related to Figure 2 and 3. Mean and standard deviation (sd) of F1-score, recall, precision and specificity of the NN predictions for all reactions and all models in the one-per-genus dataset.

	Reactions	Models
Mean F1-score	0.68 (sd = 0.29)	0.83 (sd = 0.059)
Mean recall score	0.68 (sd = 0.32)	0.84 (sd = 0.064)
Mean precision score	0.76 (sd = 0.20)	0.82 (sd = 0.074)
Mean specificity score	0.90 (sd = 0.19)	0.97 (sd = 0.010)

Supplementary Table S2: Percentage decrease and Wilcoxon ranked test of the F1-score of predictions when different phyla are excluded from training, related to Figure 3.

Firmicutes	*Actinobacteria*	*Bacteroidetes*	*Proteobacteria*
- 0.18 ($p = 2.52*10^{-66}$)	-0.24 ($p = 1.86*10^{-39}$)	-0.43 ($p = 1.01*10^{-24}$)	-0.25 ($p = 3.25*10^{-125}$)

Supplementary Table S3: Fold change of F1-score and Wilcoxon ranked test for gap-filling draft models with 30% of reactions removed using different weighting schemes, related to Figure 4.

	W1	W2	W3	W4
W1: None	x	7.29 ($p = 4*10^{-18}$)	12.87 ($p = 4*10^{-18}$)	13.98 ($p = 4*10^{-18}$)
W2: Naive binary		x	1.765 ($p = 4*10^{-18}$)	1.917 ($p = 4*10^{-18}$)
W3: Frequency-based			x	1.086 ($p = 3*10^{-18}$)
W4: NN				x

Supplementary Table S4: Mean F1-score and standard deviation (sd) for gap-filling draft-models with 30% of reactions removed using different weighting schemes, related to Figure 4.

Weights	Mean F1
W1: None	0.015849 (sd = 0.011)
W2: Naive binary	0.115597 (sd = 0.036)
W3: Frequency-based	0.203976 (sd = 0.040)
W4: NN	0.221615 (sd = 0.045)

Supplementary Table S5: Mean F1-score and standard deviation (sd) for gap-filling using different weighting schemes of 500x iML1515 with 30% of reactions removed, related to Figure 6.

Weights	Mean F1
W1: None	0.028002 (sd = 0.0073)
W2: Naive binary	0.047483 (sd = 0.0075)
W3: Frequency-based	0.156699 (sd = 0.010)
W4: NN	0.244905 (sd = 0.018)
